# Magnetically bioprinted anisotropic hydrogels promote BMSC osteogenic differentiation for bone defect repair

**DOI:** 10.1016/j.mtbio.2025.101885

**Published:** 2025-05-20

**Authors:** Rong Xu, Hua Zhang, Yang Luo, Shiyi Pan, Chi Zhang, Xiaochuan Wu, Guofeng Zhang, Cuicui Su, Dongdong Xia

**Affiliations:** a Department of Orthopedics, The First Affiliated Hospital of Ningbo University, Ningbo, 315000, Zhejiang, China; b Research Institute of Smart Medicine and Biological Engineering, Health Science Center, Ningbo University, Ningbo, 315211, Zhejiang, China; c The Second Affiliated Hospital of Anhui Medical University, Hefei, 230601, Anhui, China

**Keywords:** Anisotropic hydrogel, Bioprinting, Osteogenic differentiation, Bone repair

## Abstract

Bone tissue engineering utilizing magnetic anisotropic hydrogels (MAHs) loaded with bone marrow-derived stem cells (BMSCs) offers a promising strategy to enhance the regeneration of bone defects due to their mechanotransduction and osteoinductive properties. However, the application of MAHs as bioinks for creating personalized 3D scaffolds faces significant challenges. Bioprinting necessitates a rapid sol-gel transition to enable the ink to form stable structures, whereas anisotropic shaping requires the ink to remain in a sol state post-printing, allowing magnetic particles to assemble freely under magnetic induction. To overcome these challenges, we develop a biomimetic MAH that recapitulate the anisotropic structures of the bone using a continuous Liquid-in-Liquid bioprinting method combined with magnetic induction. The constructed MAHs feature uniformly aligned Fe_3_O_4_ microfibers embedded within the bioprinted hydrogel filaments. These Fe_3_O_4_ microfibers provide microscale geometric cues that promote the elongation and osteogenic bioactivity of BMSCs through biomechanical signaling pathways. The implantation of the MAHs loaded with BMSCs in a critical-sized cranial defect model effectively accelerates the healing of bone injuries by facilitating collagen matrix development and promoting neovascularization. This study introduces a novel approach in the development of MAHs and presents a promising candidate for applications in bone tissue engineering and repair.

## Graphical abstract

**Figure:**
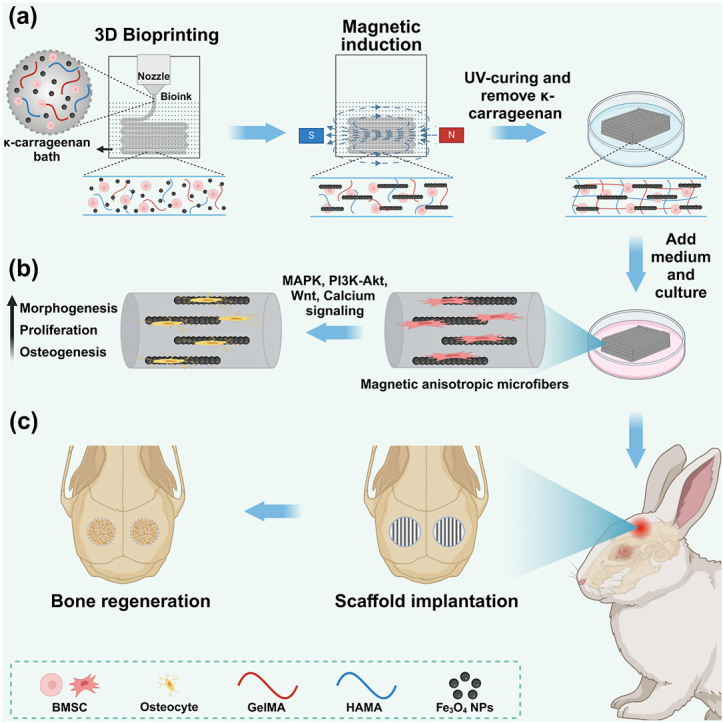

## 1 Introduction

The repair of large bone defects caused by trauma, infection, diseases, and tumors remains a considerable clinical challenge due to factors such as the complexity of the injuries and the limited availability of suitable graft materials [1]. Statistically, approximately 2.2 million patients globally require bone transplantation or replacement surgeries annually, and this demand is increasingly influenced by an aging population [2]. Current strategies for addressing these defects primarily involve surgical interventions utilizing autografts, allografts, and artificial bone substitutes [3]. The use of autografts is often constrained by donor availability, whereas allografts are associated with immune rejection issues and the potential transmission of infections [4]. Although artificial bone substitutes offer high availability and a low risk of infection, they also face significant challenges such as insufficient bone integration [5,6]. These issues highlight the urgent need for innovative and effective treatment options that could enhance healing outcomes.

Bone tissue engineering has emerged as a promising alternative, enabling the creation of functional bone structures and the reconstruction of defects through integrating stem cells and scaffolds [7]. In Particular, hydrogel scaffolds have been extensively engineered to encapsulate stem cells for bone regeneration due to their structural similarity to the extracellular matrix (ECM) [8,9]. Advances in the design of engineered hydrogels have demonstrated that various biophysical properties of the matrix, such as stiffness, stress relaxation, and topography, significantly influence cellular functions independently of biochemical cues [10]. Consequently, cells exert traction forces on these matrices, modulating their volume, membrane, and intracellular tension. These physical changes significantly influence downstream biological functions, such as stem cell differentiation, through mechanosensitive transcription factors [11,12]. Therefore, leveraging the biomechanical microenvironment to enhance cell osteogenesis for bone regeneration is particularly appealing, as it effectively mimics the endogenous physiological environment and eliminates the need for additional chemical agents.

Both bone and the periosteum surrounding cortical bone exhibit distinct anisotropic structures that are crucial for their mechanical integrity and biological functions, such as the recruitment, migration, and osteogenic differentiation of stem cells [13,14]. Research has shown that anisotropically oriented matrices, which mimic the properties of natural bone and periosteum, can significantly enhance the osteogenic differentiation of stem cells by mechanotransduction [15,16]. For instance, Li et al. demonstrated that a micro-nano hierarchical microstructure, featuring aligned channels formed through directional freeze-casting, effectively guided the migration and osteogenic differentiation of bone marrow-derived stem cells (BMSCs) [17]. Similarly, Wang et al. developed a highly anisotropic osteoconductive hydrogel composite by infiltrating sodium alginate hydrogel into the microchannels of delignified pine wood, followed by in situ mineralization of hydroxyapatite nanocrystals [18]. This composite significantly promoted osteogenic differentiation in vitro and bone formation in vivo. Despite these extensive efforts demonstrating that integrating anisotropic structures promotes cellular osteogenic differentiation on scaffold surfaces in vitro, there remains a compelling need to construct three-dimensional (3D) biomimetic anisotropic hydrogels that mechanically stimulate osteogenic transcription factors and guide microstructures closely resembling those of natural bone tissue.

3D bioprinting is increasingly employed for engineering biomimetic live constructs for repairing tissues and organs [19]. A critical factor in this process is maintaining high cell viability, which is influenced by the filament distribution in cell-loaded matrix printing, typically exceeding 150 μm [20]. However, this size poses challenges in guiding cells toward specific spatial orientations. To achieve oriented morphologies within 3D hydrogels, various shear-sensitive fillers, such as micro/nanofibers and high polymers, have been incorporated into the “inks” [[21], [22], [23]]. The bioprinting shear flow induces these fillers to form aligned microstructures, providing microscale geometric cues that promote the oriented self-organization of cells [22]. Nonetheless, these anisotropic hydrogels commonly lack osteo-inductivity, an essential property for promoting bone tissue regeneration.

Magnetic nanoparticles (MNPs), such as γ-Fe_2_O_3_ and Fe_3_O_4_, exhibit exceptional osteoinductivity and enable directional arrangement under an external magnetic field [24]. Various anisotropic scaffold structures, ranging from 2D surface modifications to 3D composites, have been developed through magnetic dipole interactions among the MNPs. For instance, Tang et al. designed a magnetic anisotropic hydrogel (MAH) by incorporating static magnetic field-induced MNPs into a polyethylene glycol diacrylate/methacrylate gelatin hydrogel, utilizing a static magnetic field to guide the assembly [25]. This MAH effectively guided the growth of BMSCs along the assembly chain of the MNPs, thereby enhancing the osteogenic bioactivity. We recently developed a Fe_3_O_4_ filament-embedded gelatin-silk fibroin composite hydrogel, constructed by integrating 3D bioprinting with magnetic induction. The bioprinted MAHs promote oriented adhesion and myogenic differentiation of BMSCs [20]. Therefore, we hypothesize that the anisotropic distribution of cyto-conducive MNPs throughout the bioprinted hydrogels would enhance the osteogenic activity of cells within the 3D matrix via mechanotransduction, ultimately facilitating bone tissue regeneration and healing. However, while preparing MAHs, maintaining a low viscosity of the ink is crucial for cell printing and the subsequent self-assembly of MNPs. This necessity undoubtedly exacerbates the conflict between low-viscosity printing and high-fidelity shaping. Furthermore, in addition to the shear forces that may damage cells during the printing process, collisions between MNPs and cells are inevitable during the assembly process, raising concerns about whether these interactions could lead to cell damage.

**Scheme 1:**
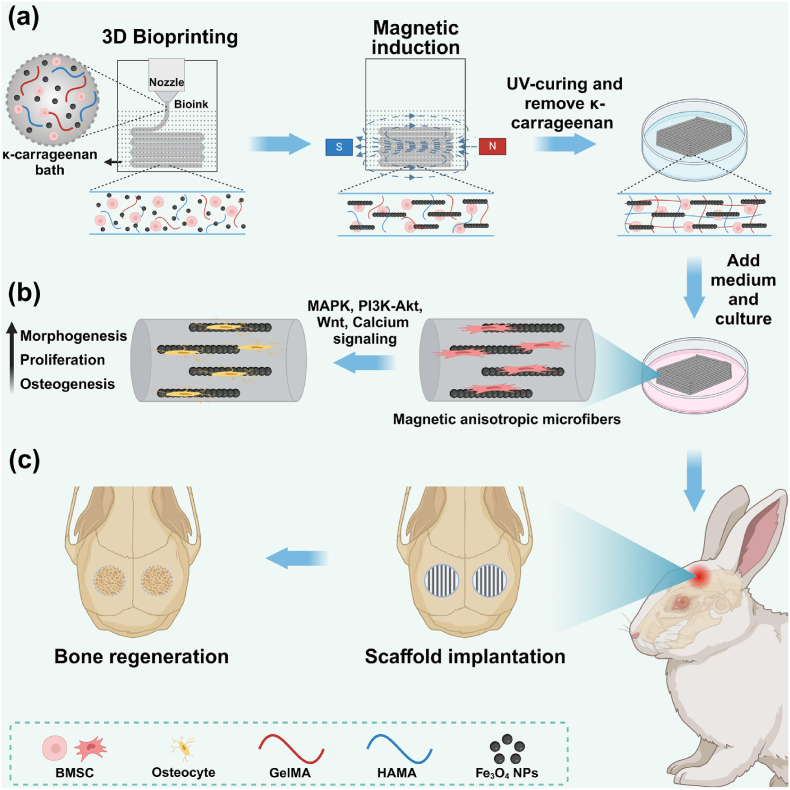
Schematic of bioprinting magnetic anisotropic hydrogels for bone repair. (a) Illustration of the preparation of magnetic anisotropy GHF hydrogel. (b) Schematic of magnetic anisotropic GHF hydrogels guiding cell orientation and promoting osteogenic differentiation. (c) Schematic illustrating the application of the magnetic anisotropic GHF hydrogel in repairing critical-sized bone defects.

To address these challenges associated with bioprinting MAHs for bone defect repair, we designed a magnetic anisotropic biomimetic hydrogel encapsulated with BMSCs using a Liquid-in-Liquid bioprinting method combined with magnetic induction. The printing bioink, referred to as GHF hydrogel, consisted of type A gelatin methacrylate (GelMA), hyaluronic acid methacryloyl (HAMA), magnetic Fe_3_O_4_ NPs, and BMSCs, which were printed in a liquid-like *κ*-carrageenan supporting bath. The synergistic combination of biocompatible and bioactive type A GelMA and HAMA enhances the continuous printability of the mixed solution in the *κ*-carrageenan bath, owing to the electrostatic interactions between them. Moreover, incorporating HAMA into GelMA strikes a balance between the mechanical stability and dynamic degradability of the hydrogels, both of which are essential for cell growth and bone tissue formation. Following bioprinting, the Fe_3_O_4_ NPs rapidly self-assemble in response to a static magnetic field, forming magnetic nanofibers aligned parallel to the direction of the magnetic field. The magnetic anisotropic GHF hydrogels were then crosslinked by 405 nm photo-initiated polymerization and could be readily removed from the supporting bath (Scheme 1a). Our results demonstrated that BMSCs loaded in the GHF hydrogel showed high cell viability exceeding 90 %. Furthermore, these cells displayed anisotropic adhesion and significantly enhanced osteogenic bioactivity, which was closely associated with biomechanical signaling pathways, including the MAPK, PI3K-Akt, Wnt, and Calcium signaling pathways (Scheme 1b). Significantly, this biomimetic GHF hydrogel, combined with BMSCs, effectively accelerated the healing of bone injuries in a critical-sized cranial defect model (Scheme 1c). Overall, the magnetic anisotropic biomimetic GHF hydrogel presents a promising candidate for bone tissue engineering and repair applications.

## 2 Materials and methods

### 2.1 Materials

Gelatin, methacrylic anhydride (MA, 94 %), caustic soda (NaOH), hyaluronic acid Lithium phenyl(2,4,6-trimethylbenzoyl)phosphinate (LAP), dialysis tubing (Mw: 8000–14000), Fe_3_O_4_ nanoparticles, and *κ*-carrageenan were purchased from Aladdin (Shanghai, China)

### 2.2 Synthesis of GelMA and HAMA

GelMA was synthesized following a previously established protocol [23]. Gelatin (50 g) was dissolved in 500 mL of phosphate-buffered saline (PBS) at 50 °C with continuous stirring. MA (10 mL) was then added at a controlled rate of 0.5 mL/min, and the reaction was allowed to proceed for 2 h under the same temperature conditions. To remove the unreacted material, the mixture was dialyzed using dialysis tubing in deionized water at 37 °C for 7 days. The GelMA solution was subsequently lyophilized and stored at −80 °C for long-term preservation.

HAMA was synthesized according to a previously reported method [26,27]. Hyaluronic acid (3 g) was dissolved in 300 mL of ultrapure water by stirring for 4 h. MA (5 mL) was then gradually added while maintaining the pH at approximately 8.0 by the dropwise addition of 10 mol/L NaOH. The reaction was conducted in the dark for 12 h. The final solution was dialyzed in ultrapure water for 3 days, lyophilized, and then stored at −80 °C for long-term preservation.

### 2.3 Characterization of HAMA and GelMA

The synthesized HAMA and GelMA were characterized using proton nuclear magnetic resonance (^1^H NMR) spectroscopy. For ^1^H NMR analysis, 5 mg of the samples were dissolved in 600 μL of deuterium oxide (D_2_O) and examined using a Bruker NMR (400 MHz, German). The degree of substitution (*DS*) for GelMA and HAMA was respectively calculated by the following equations.

$$\text{HAMA}:DS\;\left(\%\right)=\left(\frac{\left(I^{\delta H5.71}+I^{\delta H6.14}\right)/2}{I^{\delta H3.25-4.0}/10}\right)\times 100$$

$$\text{GelMA}:DS\;\left(\%\right)=\left(\frac{I^{Gel\delta H2.89}-I^{GelMA\delta H2.89}}{I^{Gel\delta H2.89}}\right)\times 100$$

### 2.4 Preparation of *κ*-carrageenan suspension bath

A suspension bath containing 0.35 % (w/v) *κ*-carrageenan was prepared according to a previously established method [28]. A quantity of 0.35 g of *κ*-carrageenan was added to 100 ml of PBS and subjected to continuous stirring at 70 °C for 30 min to ensure thorough dissolution. The solution was then incubated at 4 °C for 2 h to facilitate gel formation. To achieve a homogeneous suspension bath, the formed *κ*-carrageenan gel was continuously agitated at 1200 rpm for 1 h at ambient temperature using a top stirrer, which disrupted the gel structure. Finally, the resulting *κ*-carrageenan suspension bath was centrifuged at 1000 rpm for 3 min to eliminate entrapped air.

### 2.5 Rheology analysis

The rheological properties of the hydrogels were assessed using a rheometer (Discovery HR20, TA Instruments, USA) equipped with a 20 mm diameter plate with a 1000 μm separation. The linear viscoelastic behavior was determined through strain sweep measurements conducted at 37 °C, with shear rates ranging from 0.1 s^−1^–1000 s^−1^. The photocrosslinking process was monitored by time sweep measurements at 10 Hz and 1 % strain under 405 nm UV light. Following crosslinking, the modulus of the photocrosslinked hydrogel was evaluated through a frequency sweep at a constant strain (1 % strain; 0.1–100 rad s^−1^). To analyze the viscosity and yield stress of 0.35 % *κ*-carrageenan, a shear rate ramp in the range of 0.01–100 s^−1^ was conducted. Subsequently, to measure self-recovery behavior, the step shear tests were performed by using shear rates of 0.1 s^−1^ and 10 s^−1^ for 60 s, respectively, for three cycles.

### 2.6 Liquid-in-liquid 3D printing of GelMA/HAMA hydrogels

The bioprinting of hydrogels was conducted by our custom-designed 3D bioprinting system (Panowin-Ximai, China). The hydrogel precursor solution was deposited onto cell culture dishes (beygold, China) containing a 0.35 % (w/v) *κ*-carrageenan suspension bath to facilitate the formation of a suspended printing structure. The bioink formulation, composed of 10 % (w/v) GelMA, 0.5 % (w/v) HAMA, and 0.3 % (w/v) LAP, was supplemented with a trace amount of methyl orange to enhance visual clarity. The bioink was loaded into a 5 mL syringe equipped with a 27G needle (internal diameter: 0.21 mm), and the ambient temperature was maintained at 37 °C during the printing process. Digital models for printing were either generated using Rhino software or sourced from 3D model databases (https://www.thingiverse.com), including mesh structures, intervertebral discs, hip bones, and femur structures. These models were resized using Pango software (Panowin, China) to meet the specific printing requirements and were subsequently exported as G-code instructions to ensure accurate recognition and execution by the 3D bioprinter. The syringe containing the bioink was mounted on the 3D bioprinter, and the printing parameters were configured as follows: (1) a flow rate of 0.1 mL min^−1^, (2) a printing speed of 25 mm s^−1^, (3) a fill density of 90 %, and (4) a layer height of 0.2 mm. The Z-axis height was calibrated to ensure continuous needle immersion in the *κ*-carrageenan suspension bath throughout the entire printing process. Upon completion of printing, the constructs were exposed to 405 nm ultraviolet light for 30 s to induce photocrosslinking. Finally, the constructs were gently rinsed with PBS to eliminate the *κ*-carrageenan suspension bath.

### 2.7 Mechanical and nanoindentation testing

The mechanical properties of the hydrogels were characterized using a universal tensile testing machine (CMT1104, SASCK, China). Firstly, the GelMA/HAMA mixture solution was fabricated into cylindrical specimens with a length and diameter of 9 mm using molds or a 3D printer. Subsequently, uniaxial compression was performed at a constant rate of 10 mm min^−1^ until failure. The compression modulus was determined from the slope of the initial 10 % linear region of the resulting stress-strain curve. Each experimental group was tested in triplicate to ensure reproducibility.

The elastic modulus of the printed GelMA/HAMA hydrogels was also measured using a nanoindentation system equipped (TI-Premier, Bruker) with a Berkovich diamond indenter (radius of curvature: 48.5 μm). Following complete photopolymerization, specimens were immersed in PBS to maintain moisture during testing. A quasi-static nanoindentation protocol was implemented under force-controlled mode, achieving maximum indentation depths of 1000 nm. The testing sequence comprised three distinct phases: loading, 5-s dwell period for creep stabilization, and unloading. Continuous acquisition of load-displacement curves enabled subsequent calculation of elastic modulus through the Oliver-Pharr analysis.

### 2.8 Fabrication of magnetic anisotropic GHF hydrogels

A cubic structure hydrogel (10 × 10 × 1 mm^3^) with unidirectional lines was prepared by incorporating Fe_3_O_4_ NPs into the GelMA/HAMA hydrogel precursor solution using a 3D bioprinter. Subsequently, an N/S magnetic field was immediately applied to induce the formation of anisotropic microfibers composed of Fe_3_O_4_ NPs within the hydrogel. The hydrogel was then crosslinked using 405 nm blue light, resulting in a magnetic anisotropic hydrogel designated as AS-GHF. For comparison, a magnetic isotropic hydrogel, which was not exposed to the N/S magnetic field, was prepared and designated as IS-GHF. To investigate the influence of different Fe_3_O_4_ NP concentrations on the distribution of microfibers formed within hydrogels, we prepared three anisotropic hydrogels with Fe_3_O_4_ NP concentrations of 0.05, 0.1, and 0.2 wt%, respectively. Optical microscopy (NIB410, Nexcope, USA) was used to capture images of the hydrogels, and the longitudinal length, transverse width, and number of internal magnetic microfibers formed within each printed line at 10x magnification were quantified using ImageJ software.

### 2.9 Swelling and degradation analysis

To evaluate the swelling behavior of the hydrogels, we first weighed the prepared hydrogels (*W*_*0*_) and then immersed them in PBS at 37 °C. During the testing period, fresh PBS was replenished every 2 days. Once the hydrogels reached equilibrium swelling, the surface water was removed, and the hydrogels were reweighed (*W*_*t*_). The swelling ratio was then determined using the following formula (n = 3):

$$\text{Swelling}\;\text{ratio}\;\left(\%\right)=\frac{W_{t}-W_{0}}{W_{0}}\times 100$$

The in vitro degradation behavior of the hydrogel was assessed by monitoring the mass change of freeze-dried hydrogel scaffolds in PBS. Initially, the freeze-dried hydrogels were weighed, and the initial mass (*M*_*0*_) was recorded. The scaffolds were then incubated in PBS at 37 °C, with the PBS solution being refreshed every two days. At designated time points, the scaffolds were retrieved, freeze-dried, and reweighed to obtain the mass (*Mᵢ*). The degradation rate of the hydrogel was calculated using the following formula (n = 3):

$$\text{Degradation}\;\text{rate}\;\left(\%\right)=\frac{M_{0}-M_{i}}{M_{0}}\times 100$$

### 2.10 Rabbit bone mesenchymal stem cells (BMSCs) isolation and culture

Female New Zealand White Rabbits (3 weeks) were obtained from Ningbo University (Ningbo, China). The bone marrow was extracted from the tibial and femoral bones according to established protocols [29]. Briefly, the femurs and tibias were excised, and the bone marrow was flushed out using basal medium. The collected cells were centrifuged and subsequently resuspended in 1640 medium (RPMI1640, Vivacell Biosciences) supplemented with 10 % fetal bovine serum (FBS, Vivacell Biosciences) and 1 % penicillin/streptomycin (NCM biotech, China). The cells were cultured at 37 °C in a humidified atmosphere containing 5 % CO_2_. Upon reaching 80 % confluence, the cells were trypsinized and passaged. Cells from the 4th to 6th generation were used for subsequent experiments.

### 2.11 Fabrication of magnetic anisotropic GHF hydrogels loaded with BMSCs

The lyophilized GelMA and HAMA were exposed to ultraviolet light for 30 min for sterilization. Subsequently, BMSCs at a concentration of 10^7 cells per mL were resuspended in a GelMA/HAMA solution containing 0.2 wt% Fe_3_O_4_ NPs at 37 °C and transferred into a 5 mL syringe [30]. Magnetic anisotropic hydrogels (AS-GHF + BMSCs) and magnetic isotropic hydrogels (IS-GHF + BMSCs) with dimensions of 10 × 10 × 1 mm^3^ were fabricated using the printing technique previously described. The hydrogels were then cultured in 1640 medium supplemented with 10 % FBS and 1 % penicillin/streptomycin to support further growth and development. To eliminate potential confounding effects of the N/S magnetic field used for Fe_3_O_4_ NPs induction on BMSCs within hydrogels, we incorporated BMSCs into GelMA/HAMA bioink and fabricated hydrogel constructs with dimensions of 10 × 10 × 1 mm^3^ using bioprinting technology. The composite hydrogels were subsequently exposed to the identical N/S static magnetic field treatment protocol, followed by photopolymerization and standard culture maintenance.

### 2.12 Live/Dead assay and cytoskeleton staining assay

Cell viability in hydrogels was detected by Live/Dead staining. In brief, the cells within the hydrogels were stained with a calcein-AM/PI solution (Beyotime, China) after 1 day of culture and visualized using a Laser Scanning Confocal Microscope (LSCM, STELLARIS 5, Leica, Germany).

To observe the growth morphology of BMSCs, cytoskeletal staining was performed. After 3 and 5 days of culture, the samples were fixed with 4 % paraformaldehyde for 1 h and permeabilized with 0.5 % Triton X-100 (Beyotime, China) for 30 min. The samples were then stained with TRITC-phalloidin (1:300, Solarbio, China) at room temperature, protected from light, for 1 h. Nuclei were counterstained with DAPI (Solarbio, China), and images were acquired using the LSCM. ImageJ software was used to analyze the images and quantify the length and orientation angle distribution of the long axis of the cytoskeleton.

### 2.13 In vitro osteogenic differentiation assay

To evaluate the osteogenic differentiation potential of BMSCs in vitro, the samples were cultured in an osteogenic differentiation induction medium. Immunofluorescence staining was conducted on the cells at 7 and 14 days. Following three washes with PBS, cells were fixed and permeabilized for 1 h each and then blocked with 3 % BSA (Beyotime, China) for 6 h. Subsequently, cells were incubated with primary antibodies against Osteopontin (OPN, 1:200, Proteintech, USA, 22952-1-AP) overnight at 4 °C. The corresponding goat anti-rabbit secondary antibody (1:1000, Alexa Fluor® 488, Abcam, UK, ab150077) was applied at room temperature for 6 h. After three PBS washes, cells were stained with TRITC-phalloidin (1:300) for 1 h and DAPI for 30 min. Cell morphology was visualized using the LSCM and analyzed with ImageJ software.

### 2.14 RNA sequencing and analysis

Total RNA was extracted using TRIzol reagent (Invitrogen, CA, USA) according to the manufacturer's protocol. RNA purity and quantification were evaluated using the NanoDrop 2000 spectrophotometer (Thermo Scientific, USA). RNA integrity was assessed using the Agilent 2100 Bioanalyzer (Agilent Technologies, Santa Clara, CA, USA). Then the libraries were constructed using VAHTS Universal V6 RNA-seq Library Prep Kit according to the manufacturer instructions. The transcriptome sequencing and analysis were conducted by OE Biotech Co., Ltd. (Shanghai, China).

### 2.15 Quantitative real-time PCR analysis

Following 14 days of culture, total RNA was extracted from BMSCs within the hydrogel using TRIzol Reagent (TransGen, China). The isolated RNA was reverse transcribed into cDNA utilizing the EasyScript® All-in-One First-Strand cDNA Synthesis SuperMix for qPCR kit (TransGen, China). Quantitative real-time PCR (qRT-PCR) was then conducted using the PerfectStart® Green qPCR SuperMix kit (TransGen, China) on a LightCycler 480 Instrument II (Roche, Switzerland). Relative gene expression levels were determined using the 2^−△△Ct^ method, normalized to the Ct value of the housekeeping gene (GAPDH). The primer sequences are shown in Table 1. Three parallel samples were prepared for each experimental group.

**Table 1:** Primers used for RT-qPCR.

Gene	Forward sequence	Reverse sequence
GARDH	CCACTTTGTGAAGCTCATTTCCT	TCGTCCTCCTCTGGTGCTCT
BGLAP	CTCACTCTTGTCGCCCTGCT	TCTTGGACACGAAGGCTGAG
COL1A1	AGGGCGACAGAGGCATAAAG	GCCGTTGAGTCCATCTTTGC
BMP6	ACCCCACAGCATAACATGGG	TGAAGGGCTGCTTGTCGTAG
IGF1	CATCCTGTCCTCCTCGCATC	CCGTATCCTGTGGGCTTGTT

### 2.16 Hemolysis assay

The blood compatibility of hydrogels was evaluated using whole blood collected from an 8-week-old New Zealand rabbit [31]. Briefly, 0.1 mL of blood was added to 10 mL of saline or deionized water, serving as the negative and positive control groups, respectively. The AS-GHF hydrogel was introduced into an additional negative control group. All samples were incubated in a 37 °C water bath for 60 min and subsequently centrifuged at 2000 rpm min^−1^ for 15 min. The absorbance of the suspension at 545 nm was measured using a spectrophotometer, with the procedure being performed in triplicate. The hemolysis ratio (*HR*) was calculated using the following formula:

$$HR\left(\%\right)=\frac{{OD}_{Sample}-{OD}_{Negative}}{{OD}_{Positive}-{OD}_{Negative}}\times 100$$

where *OD*_*Sample*_, *OD*_*Negative*_ and *OD*_*Positive*_ stand for the optical density of hydrogel, saline, and 0.1 % Triton-X-100 solution, respectively.

### 2.17 In vivo bone defect repair

Animal experiments were conducted with the approval of the Animal Ethics and Welfare Committee (AEWC) of Ningbo University, under institutional ethics clearance (approval number: 12492). The in vivo osteogenic potential of AS-GHF hydrogels was assessed using critical-sized calvarial defect models in New Zealand White Rabbits. In brief, the skulls were exposed, and two critical-sized defects (7 mm) were created using a ring drill. The bone defects were randomly allocated into three groups (n = 3): control, AS-GHF, and AS-GHF + BMSCs. The corresponding scaffolds were implanted in the AS-GHF group and the AS-GHF + BMSCs group, respectively. The control group received no implants, leaving the defects untreated. Resorbable sutures were used to close the wounds, and postoperative care included intramuscular injections of 10^4^ U of penicillin sodium for three days to prevent infection.

Additionally, a subcutaneous implantation experiment was performed to evaluate the in vivo degradation and inflammatory response of the AS-GHF hydrogel. Eight New Zealand White Rabbits were selected, and four injection sites were marked subcutaneously on their backs. Each site received 0.2 mL of hydrogel and light-cured for 2 min, with an interval of 2 cm between each site. Tissue samples encapsulating the injected hydrogels were collected at four time points (3, 7, 14, and 21 days) for subsequent histopathological analysis.

### 2.18 Micro-CT analysis

Calvarial tissues from all experimental New Zealand White Rabbits were harvested at 6 and 12 weeks postoperatively and subsequently fixed in 4 % paraformaldehyde at room temperature for 48 h. Complete skulls were subjected to micro-computed tomography (micro-CT, Duper Nova, PINGSENG Healthcare Inc., China). Following the scanning process, three-dimensional images were reconstructed, and key parameters, including bone volume fraction (BV/TV), bone mineral density (BMD), trabecular thickness (Tb.Th), and trabecular separation (Tb.Sp) were quantified using the micro-CT system's auxiliary software.

### 2.19 Histological and immunohistochemical analysis

At 6 and 12 weeks, the skull tissues were harvested and fixed in 4 % paraformaldehyde for 48 h, followed by decalcification in an EDTA solution. Post-decalcification, the samples were embedded in paraffin and sectioned into 5 μm slices. Furthermore, the hearts, livers, spleens, lungs and kidneys were collected at 12 weeks for long-term biosafety assessment. The sections were stained using standard Hematoxylin and Eosin (H&E) and Masson's trichrome staining. Immunohistochemical (IHC) staining for OPN and CD31 (1:2000, proteintech, USA, 11265-1-AP) was conducted on bone tissue sections to assess the osteogenic and angiogenic activities associated with different treatment groups.

### 2.20 Statistical analysis

All experiments were performed in three independent studies and data were expressed as means ± standard deviations. One-way analysis of variance (ANOVA) followed by Tukey's post hoc test or unpaired Student's t-test, two-tailed, was used to analyze the statistical significance where appropriate. Significant difference was considered at ∗p < 0.05, ∗∗p < 0.01, ∗∗∗p < 0.001 and ∗∗∗∗p < 0.0001.

## 3 Results and discussion

### 3.1 Liquid-in-liquid printing of GelMA/HAMA composites

The polymer matrix of the magnetic anisotropic biomimetic GHF hydrogels is composed of cationic type A GelMA and anionic HAMA, which can form hydrogen bonds and electrostatic interactions between them, crucial for maintaining the integrity of the liquid-printed structures. GelMA and HAMA were synthesized by grafting methacrylic anhydride onto gelatin and hyaluronic acid (HA) (Fig. S1a). The chemical structures of GelMA and HAMA were validated using proton nuclear magnetic resonance (^1^H NMR), revealing a methacrylate substitution degree of approximately 62 % for GelMA and 23 % for HAMA (Fig. S1b). In order to achieve optimal printing fluidity and anti-diffusion capability of the hydrogel precursor, along with promoting the dynamic degradation behavior necessary for cellular growth and long-term stability in bone tissue regeneration, a 0.5 % (w/v) HAMA was introduced into a 10 % (w/v) GelMA solution to prepare the hydrogel matrix.

**Fig. 1:**
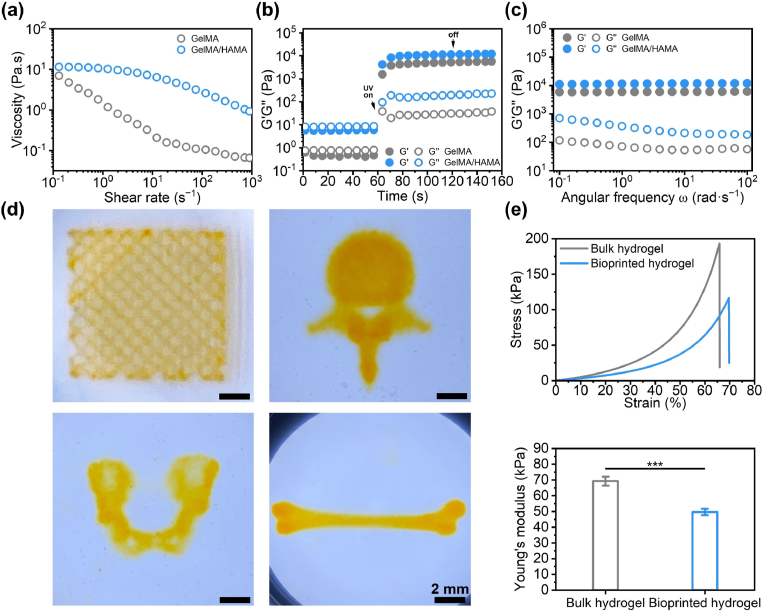
Rheological and mechanical characterization of GelMA/HAMA hydrogels. (**a-c**) Shear rate (**a**), time (**b**), and frequency (**c**) sweeps for GelMA/HAMA and GelMA hydrogels at 37 °C. (**d**) Biomimetic printed structures made from GelMA/HAMA hydrogels. (**e**) Compressive stress-strain curves and Young's moduli for bioprinted and bulk hydrogels. (n = 3, ∗∗∗p < 0.001).

The viscosity and gelation kinetics of GelMA/HAMA at 37 °C were initially examined, using GelMA as a comparison. The incorporation of HAMA resulted in a slight increase in the viscosity of the GelMA/HAMA solution while maintaining a constant zero-shear viscosity between 0.1 and 1 s^−1^, suggesting effective formation of hydrogen bonds and electrostatic interactions between GelMA and HAMA (Fig. 1a). Moreover, GelMA and GelMA/HAMA solutions exhibited decreased viscosity with increasing shear rates; however, the GelMA/HAMA solutions displayed a more gradual shear-thinning behavior compared to GelMA. These properties enhance the injectability during micro-extrusion printing and contribute positively to the structural integrity of the printed constructs post-printing. Upon exposure to 405 nm blue light irradiation, both GelMA and GelMA/HAMA underwent a rapid fluid-to-gel transition, occurring within 19 s and reaching a stable modulus within 25 s (Fig. 1b). Due to the chemical cross-linking between GelMA and HAMA, the GelMA/HAMA hydrogel displayed a higher storage modulus (G′) of 11.5 kPa compared to 5.2 kPa for the GelMA hydrogel. These G’ values remained constant over a frequency range of 0.1–100 rad s^−1^, indicating the formation of a highly elastic network (Fig. 1c).

Owing to the efficient hydrogen bonding and electrostatic interactions between GelMA and HAMA, the GelMA/HAMA composite solution exhibits good fluidity and anti-diffusion properties. However, its self-supporting capability is low when directly used for micro-extrusion printing. The utilization of the thermosensitive sol-gel transition properties of GelMA/HAMA allows for enhanced fidelity in printed microstructures; nonetheless, a rapid decrease in temperature adversely affects cell viability. Therefore, to achieve high-fidelity solution bioprinting with high cytocompatibility, an optimized *κ*-carrageenan supporting bath was utilized basing on our prior investigation [32]. Compared to microgel support baths made from gelatin [33], agarose [34], and gellan gum [35], etc., this *κ*-carrageenan bath exhibited a low yield stress of 0.008 Pa at 37°C, along with sensitive shear-thinning and rapid self-healing behavior, which are essential for maintaining the integrity of printed structures (Fig. S2). Additionally, as an anionic polysaccharide, *κ*-carrageenan interacts with the charges present in type A GelMA, aiding in temporarily maintaining the prints in suspension. To ensure the printing ink and the suspension remained in a low-viscosity solution, we maintained the printing temperature at 37 °C, at which the *κ*-carrageenan bath and GelMA/HAMA bioink exhibited excellent flowability (Fig. S3, Video S1).

Utilizing this Liquid-in-Liquid printing technology, various constructs were successfully fabricated, including grid, intervertebral discs, hip bone, and femoral. As shown in Fig. 1d, all printed models maintained structural integrity without observable collapse, while preserving fine structural details critical. These hydrogel constructs demonstrate that the *κ*-carrageenan liquid suspension bath provided high-fidelity printing support for the GelMA/HAMA solution. Therefore, the GelMA/HAMA composite matrix fulfills the prerequisites for the preparation of anisotropic hydrogels through Liquid-in-Liquid printing technology. The printed GelMA/HAMA hydrogels, containing 90 % filler, exhibited high anti-compressive strength and modulus, with values reaching 116 kPa and 49.7 kPa, respectively (Fig. 1e). Although the mechanical properties of the bioprinted hydrogels were lower than those of bulk hydrogels (strength and modulus: 193 kPa and 69.2 kPa), they still provided sufficient self-supporting capability for the printed structures. Additionally, the elastic modulus of the printed GelMA/HAMA hydrogels was measured by using nanoindentation, yielding a value of 52.72 ± 2.28 kPa (Fig. S4), which is consistent with the compressive modulus. This modulus not only maintains the structure of the printed scaffold but is also beneficial for the growth and osteogenic differentiation of stem cells in three-dimensional space [36].

### 3.2 Construction of anisotropic hydrogels by liquid-in-liquid bioprinting

**Fig. 2:**
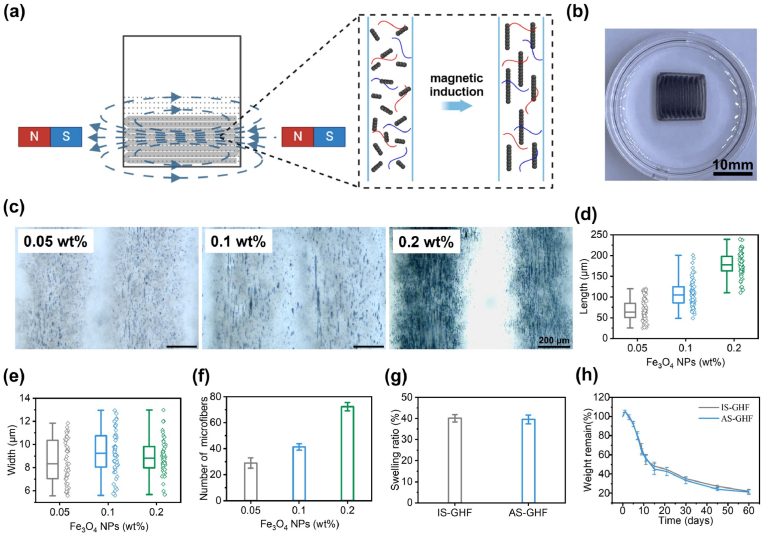
Microstructural characterization of anisotropic hydrogels. (**a**) Magnetic induction orientation diagram. (**b**) Top view of the anisotropic hydrogel. (**c**) Optical microscope images of micro-nano stripes formed by the magnetic induction orientation of Fe_3_O_4_ NPs with different concentrations. (**d-f**) Length (**d**), width (**e**), and number (**f**) of microfibers formed by different concentrations of Fe_3_O_4_ NPs. (**g**) Equilibrium swelling ratios and (**h**) degradation rates of isotropic hydrogels and anisotropic hydrogels. (n = 3).

The anisotropic hydrogel (AS-GHF) was fabricated by bioprinting GHF composites with the assistance of a static magnetic field (Fig. 2a). In contrast, only the bioprinted GHF hydrogel was applied to fabricate the corresponding isotropic hydrogel (IS-GHF). To determine the optimal concentration of Fe_3_O_4_ NPs for AS-GHF hydrogels, Fe_3_O_4_ NPs at 0.05, 0.1, and 0.2 wt% were incorporated into the GelMA/HAMA solutions. These GHF inks were printed in the *κ*-carrageenan suspension bath and subsequently subjected to a static magnetic field to induce the self-assembly of Fe_3_O_4_, forming oriented microstructures. The printed constructs were then photocrosslinked to fix the Fe_3_O_4_ microstructures within the GHF hydrogels (Fig. 2b). Under the static magnetic field, the Fe_3_O_4_ NPs rapidly aligned into self-assembled microfibers within the printed GHF gels (Video S2). These magnetic microfibers increased proportionally with Fe_3_O_4_ concentrations, and at 0.2 wt%, uniformly distributed Fe_3_O_4_ fibers were observed throughout the scaffold (Fig. 2c). Specifically, as Fe_3_O_4_ NP concentrations increased from 0.05 wt% to 0.1 wt% and 0.2 wt%, the corresponding length of magnetically induced microfibers increased, measuring 68.17 ± 26.06 μm, 107 ± 31.68 μm, and 178.64 ± 27.03 μm, respectively (Fig. 2d). The corresponding widths of Fe_3_O_4_ fibers were 7.08 ± 0.88 μm, 9.32 ± 1.94 μm, and 8.96 ± 1.49 μm, respectively, with no significant differences (Fig. 2e). Additionally, statistical analysis further demonstrated that higher Fe_3_O_4_ NP concentrations produced more microfibers and uniform fiber lengths across samples (Fig. 2f, Fig. S5). These results suggest that 0.2 wt% Fe_3_O_4_ NPs are optimal for generating uniform and sufficiently long magnetic fibers within the scaffold. Therefore, 0.2 wt% Fe_3_O_4_ NPs were used for subsequent research.

The printed AS-GHF hydrogels exhibited favorable stability and degradability in the liquid phase, which are crucial factors for supporting cell growth and promoting tissue regeneration. To assess the stability of the printed hydrogels, we evaluated their swelling behavior in phosphate-buffered saline (PBS). The swelling ratios for the IS-GHF and AS-GHF hydrogels were measured at 40.13 ± 1.8 % and 39.98 ± 2.1 %, respectively, with no significant differences observed (Fig. 2g). These low swelling ratios indicated that the GHF hydrogels possessed a stable network structure, primarily due to the synergistic effects and covalent linkages between GelMA and HAMA. Such stability is critical for maintaining the integrity of printed structures. Furthermore, degradability is an essential characteristic in bone tissue engineering, as ideal materials should exhibit degradation over a period of at least two months to align with the pace of bone regeneration. After two months in PBS, the AS-GHF and IS-GHF hydrogels retained 21.24 ± 2.4 % and 22.13 ± 2.2 % of their original mass, respectively, which adequately meets the degradation requirements for scaffolds used in bone tissue engineering (Fig. 2h). These results suggest that AS-GHF hydrogels have significant potential for cell encapsulation and bone tissue implantation applications due to their favorable stability and controlled degradability.

### 3.3 Anisotropic GHF hydrogels potentiate BMSCs alignment and osteogenesis

**Fig. 3:**
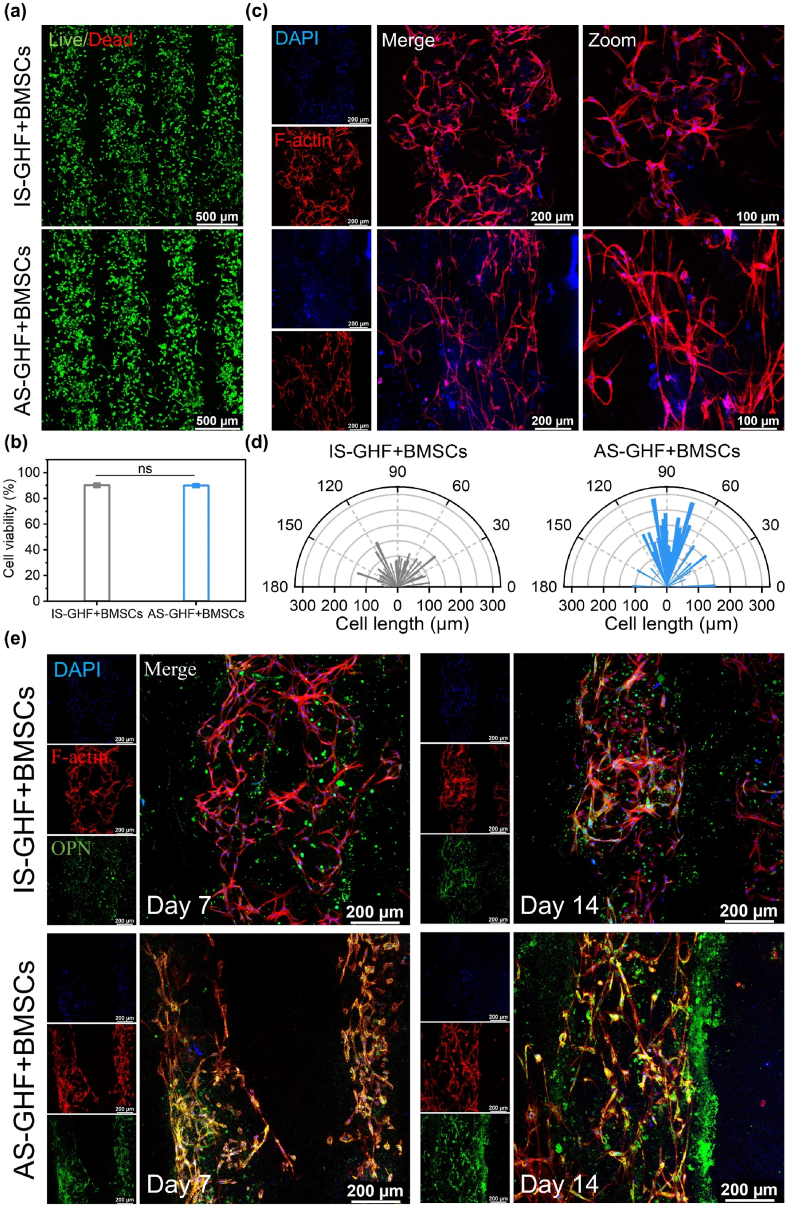
In vitro cytocompatibility, cell morphology analysis and osteogenic performance. (**a, b**) Representative Live/Dead staining images (**a**) and quantitative analysis (**b**) of BMSCs within IS-GHF and AS-GHF hydrogels after 1 day of culture. (**c**) Representative images of BMSC morphologies within IS-GHF and AS-GHF hydrogels following 5 days of culture. (**d**) Analysis of cytoskeletal length and angle distribution, with the printing direction oriented at 90°. (**e**) Immunofluorescence staining images of osteopontin (OPN) after 7 and 14 days of osteogenic induction. (n = 3).

To investigate the ability of GHF magnetically printed hydrogels to induce the BMSC alignment within 3D hydrogels, rabbit-derived BMSCs were encapsulated in the GHF precursor bioink for bioprinting. Cell viability was initially assessed using Live/Dead staining (Fig. 3a). On the first day, a significant number of viable cells were observed in both the IS-GHF and AS-GHF hydrogels, with survival rates of viable cells at 90.1 % and 89.9 %, respectively (Fig. 3b). This indicates that the GHF hydrogels produced via magnetic bioprinting did not affect cell viability. Furthermore, after 3 and 5 days of BMSC culture, the morphologies of BMSCs within the printed hydrogels were examined using rhodamine-phalloidin and DAPI staining (Figure S6, Fig. 3c). BMSCs in the AS-GHF hydrogels exhibited high elongation in alignment with the magnetic Fe_3_O_4_ microfibers, while cells in the IS-GHF hydrogels spread randomly. Quantitative analysis revealed that the majority of cells in the AS-GHF hydrogel not only displayed cytoskeletal orientation at angles of approximately 90° but also had an average cellular length of 150.25 ± 51.76 μm. This length is approximately 1.86 times greater than that observed in the IS-GHF hydrogels (80.81 ± 30.64 μm) (Fig. 3d). These results demonstrate that the magnetically assisted bioprinted AS-GHF hydrogels significantly enhance cell alignment and increase cytoskeleton length, which may facilitate osteogenic differentiation.

To validate the osteogenic potential of magnetic anisotropic biomimetic GHF hydrogels on BMSCs, the expression of osteogenesis-related osteopontin (OPN) proteins in cells was evaluated after 7 and 14 days of osteogenic induction culture. OPN was labeled with green fluorescence, F-actin was labeled with red fluorescence, and nuclei were labeled with blue fluorescence. As shown in Fig. 3e, BMSCs encapsulated within the AS-GHF hydrogels demonstrated significantly elevated levels of OPN at both 7 and 14 days compared to those in the IS-GHF hydrogels. Quantitative analysis indicated that the mean fluorescence intensity of OPN in the AS-GHF hydrogels was 2.34 times higher at 7 days and 1.59 times higher at 14 days compared to that observed in the IS-GHF hydrogels (Fig. S7). These results suggest that the AS-GHF hydrogel effectively promotes the osteogenic differentiation of BMSCs, which is essential for bone regeneration. Additionally, to investigate the influence of the static magnetic field used during the printing process on the growth and differentiation of BMSCs, we prepared GelMA/HAMA hydrogels subjected to the same static magnetic field conditions, serving as a control group. The results indicate that, BMSCs embedded within the GelMA/HAMA hydrogel exposed to the static magnetic field exhibited no significant differences in viability, growth status, and osteogenic differentiation of BMSCs within the hydrogel matrix when compared to the BMSCs within IS-GHF (Fig. S8). These findings suggest that the enhanced osteoinductive capability observed in the AS-GHF can be attributed to the anisotropic magnetic microfibers, rather than the magnetic field itself.

### 3.4 Osteogenic mechanism of BMSCs in anisotropic GHF hydrogels

**Fig. 4:**
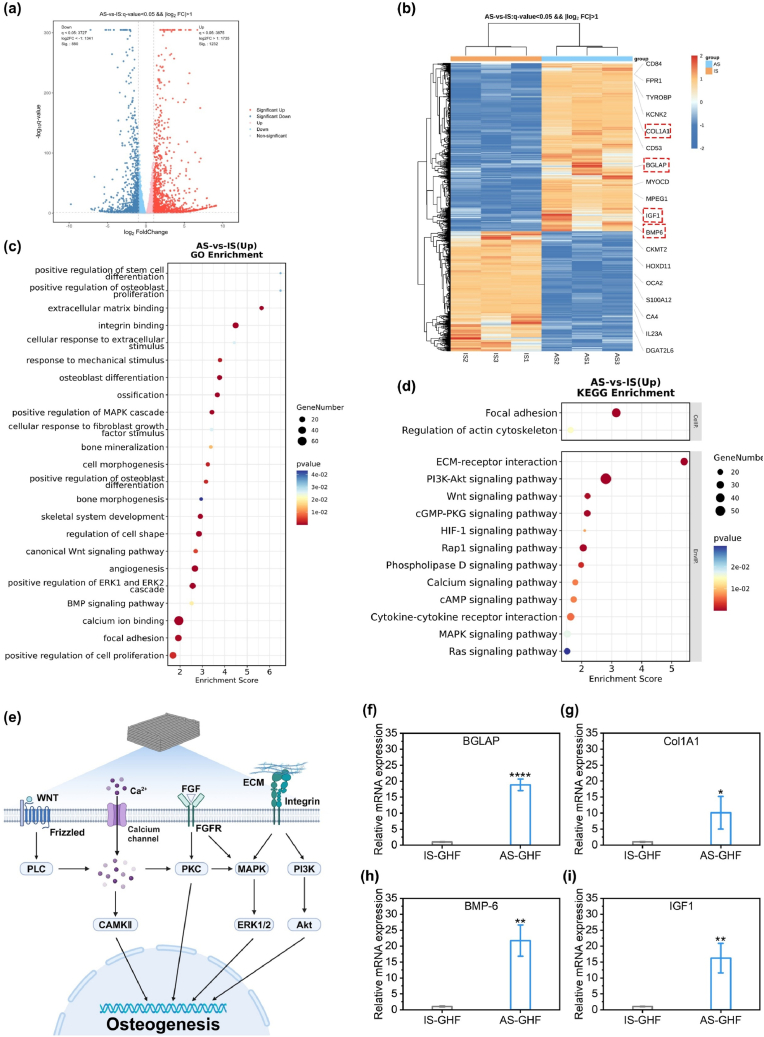
RNA sequencing analysis of BMSCs cultured within IS-GHF and AS-GHF hydrogels. (**a, b**) Volcano plot (**a**) and heatmap analysis (**b**) of differentially expressed genes, with up-regulated genes marked in red and down-regulated genes in blue. Cutoff criteria: *q*-value <0.05 and |log_2_ FC| > 1. (**c, d**) Significantly enriched GO terms (**c**) and KEGG pathways (**d**). (**e**) Schematic representation of the osteogenic differentiation mechanism of BMSCs within AS-GHF hydrogels. (**f**–**i**) Expression levels of osteogenic-related genes in BMSCs cultured within IS-GHF and AS-GHF, including BGLAP, COL1A1, BMP6, and IGF1. (n = 3, ∗p < 0.05, ∗∗p < 0.01, ∗∗∗p < 0.001 and ∗∗∗∗p < 0.0001). (For interpretation of the references to colour in this figure legend, the reader is referred to the Web version of this article.)

To investigate the osteogenic mechanism of AS-GHF hydrogels on BMSCs, bioinformatics analysis of RNA transcriptome sequencing was conducted, using IS-GHF hydrogels as the control group. Differential expression analysis, along with Gene Ontology (GO) functional enrichment and Kyoto Encyclopedia of Genes and Genomes (KEGG) pathway-enrichment analyses, was performed to explore the transcriptional profile differences and potential biological functions between the AS-GHF and IS-GHF groups. Principal component analysis (PCA) showed a markedly distinct distribution of components between the AS-GHF and IS-GHF groups (Fig. S9). The volcano plot identified a total of 880 upregulated and 1232 downregulated differentially expressed genes (DEGs) based on a q-value <0.05 and |log_2_ FC| > 1 (Fig. 4a). A heatmap visually represented the expression patterns of these DEGs (Fig. 4b). GO enrichment analysis highlighted significant biological functions associated with the DEGs, including integrin binding, ERK1/2 cascade, osteoblast differentiation, cell shape regulation, response to mechanical stimulus, extracellular matrix binding, cellular response to fibroblast growth factor (FGF), and positive regulation of cell proliferation (Fig. 4c). Furthermore, KEGG pathway analysis was conducted to identify the key regulatory signaling pathways involved in the osteogenic process. As shown in Fig. 4d, the DEGs were primarily implicated in several critical pathways, including the PI3K-Akt signaling pathway, Wnt signaling pathway, mitogen-activated protein kinase (MAPK) signaling pathway and Calcium signaling pathway.

The mechanism by which magnetic anisotropic hydrogels activate these signaling pathways may involve the activation of cellular mechanotransduction through contact guidance effects. Previous studies have shown that the extracellular matrix-integrin-cytoskeletal axis mediates the transduction of biomechanical signals into cellular biochemical signals, which is essential for the maintenance and regeneration of osteocytes [37]. The upregulation of the FGF signaling pathway is also closely associated with bone formation [38]. Therefore, they may collectively activate the MAPK signaling pathway and the ERK1/2 cascade, both of which are critical signaling pathways for mechanotransduction and osteoblast differentiation [39,40]. Additionally, magnetic anisotropic hydrogels may activate the PI3K-Akt, Wnt and Calcium signaling pathways in BMSCs through their mechanical microenvironment and the micro-magnetic sources generated by Fe_3_O_4_, which play a crucial role in facilitating osteogenic differentiation of BMSCs [[41], [42], [43]]. The proposed mechanisms are illustrated in Fig. 4e. These sequencing results were validated by qRT-PCR, which confirmed significantly higher expression levels of all four osteogenesis-related genes (COL1A1, BGLAP, BMP-6, and IGF1) in the scaffold group compared to the control group (Fig. 4f–i). These findings highlight notable advancements in bone formation facilitated by the scaffold. Therefore, anisotropic GHF hydrogel scaffold demonstrates a pronounced osteogenic effect.

### 3.5 Bone regeneration of anisotropic GHF hydrogels

The AS-GHF hydrogels facilitated the osteogenic differentiation of BMSCs by providing biomechanical stimuli independently of chemical cues, which presents substantial potential for application in bone defect repair. To ensure hemolysis and inflammatory response in vivo during implantation, we measured the hemocompatibility and histocompatibility by using a hemolysis assay and subcutaneous implantation. The hemolysis rate was less than 5 %, indicating that the AS-GHF hydrogels had good hemocompatibility (Fig. S10). Moreover, almost no inflammatory cells were observed after 21 days of implantation in New Zealand White Rabbits (Fig. S11). Consequently, the AS-GHF hydrogel exhibits good biocompatibility, satisfying the prerequisite conditions for implantation in bone defects in vivo.

**Fig. 5:**
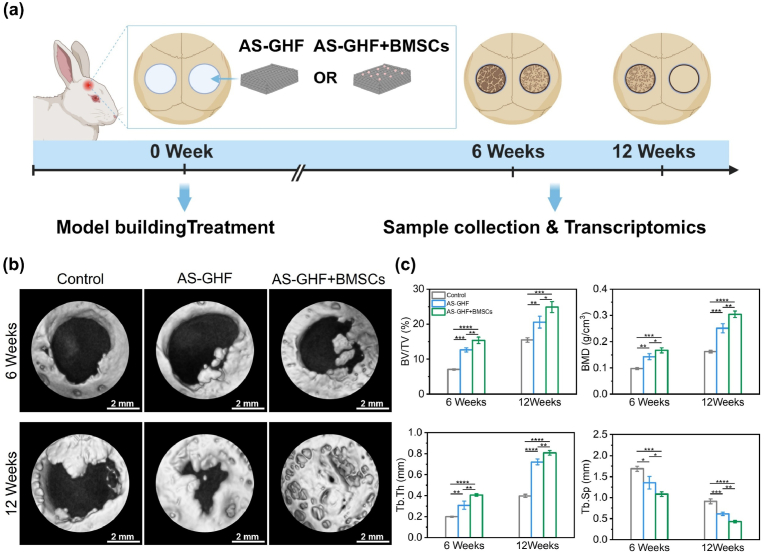
The in vivo repair effects of AS-GHF and AS-GHF + BMSCs on Calvarial defects in rabbits. (**a**) Experimental schematic diagram of AS-GHF and AS-GHF + BMSCs repairing critical size bone defect of rabbit skull. (**b**) 3D micro-CT reconstruction images of rabbit Calvarial Defect Models at 6 and 12 weeks after implantation. (**c**) The results of micro-CT quantitative analysis (BV/TV, BMD, Tb.Th, Tb.Sp) in different groups. (n = 3, ∗p < 0.05, ∗∗p < 0.01, ∗∗∗p < 0.001 and ∗∗∗∗p < 0.0001).

To assess the in vivo capability of the AS-GHF hydrogels encapsulated with BMSCs for repairing critical-sized bone defects, a 7 mm cranial defect was surgically induced in New Zealand White Rabbits. Cranial samples were collected at 6 and 12weeks post-implantation, and new bone formation was evaluated using micro-CT imaging (Fig. 5a). At 6-weeks mark, micro-CT analysis revealed minimal new bone formation at the defect margins in the control group; however, the AS-GHF and AS-GHF + BMSCs groups exhibited more substantial bone regeneration (Fig. 5b). By 12 weeks, a significant increase in newly formed bone extending toward the defect center was observed in the AS-GHF and AS-GHF + BMSCs groups compared to the control group. Notably, the AS-GHF + BMSCs group demonstrated superior new bone formation relative to the GHF group at both time points, culminating in complete bone bridging by 12 weeks. Quantitative analysis of micro-CT data including bone volume fraction (BV/TV), bone mineral density (BMD), trabecular thickness (Tb.Th), and trabecular separation (Tb.Sp) was performed (Fig. 5c). The results demonstrated that at 6 and 12 weeks, the BV/TV in the AS-GHF group was significantly greater compared to the control group, indicating enhanced osteogenic activity of AS-GHF hydrogel. Moreover, the BV/TV in the AS-GHF + BMSCs group was markedly higher than that observed in the AS-GHF group at 6 weeks (15.37 ± 0.93 % vs. 12.66 ± 0.62 %, *p* < 0.01) and at 12 weeks (24.88 ± 1.54 % vs. 20.58 ± 1.69 %, *p* < 0.05). The Tb.Th and Tb.Sp supported the trends observed in the BV/TV analysis, further confirming the efficacy of the AS-GHF + BMSCs group. This improvement in bone regeneration implies a synergistic osteogenic effect facilitated by the integration of the magnetic anisotropic hydrogel and BMSCs. Additionally, we collected the major organs (heart, liver, spleen, lungs, and kidneys) of rabbits implanted for 12 weeks to assess the long-term biosafety of AS-GHF hydrogels. H&E staining revealed no tissue abnormalities, such as cell necrosis or structural damage, in these vital organs (Fig. S12). These results further confirm the biocompatibility of the hydrogels within the host organism.

**Fig. 6:**
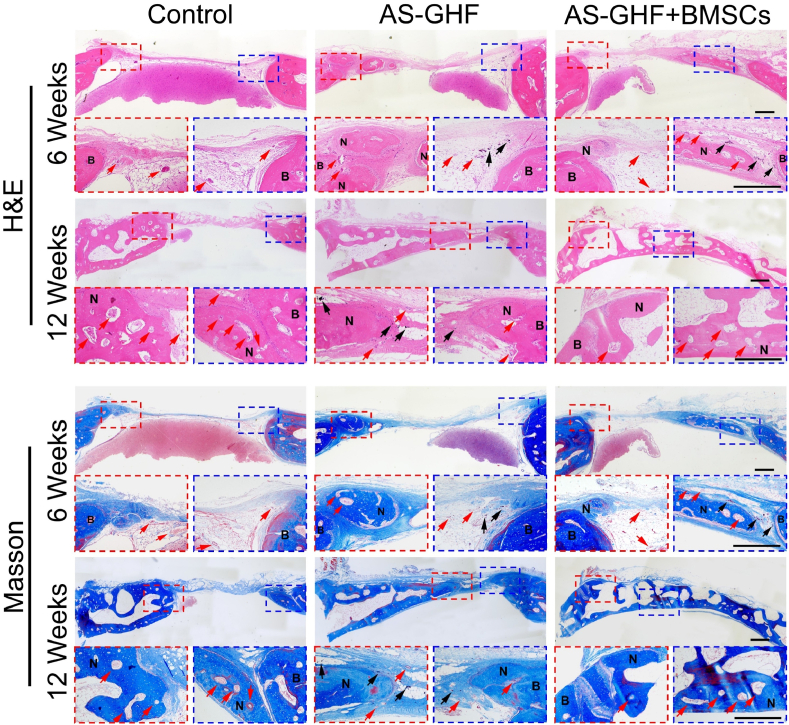
H&E staining and Masson staining of newly formed bone were performed at 6 and 12 weeks after operation. (NB: new bone; HB: host bone; Red arrow: new blood vessels; Black arrow: Fe_3_O_4_ NPs. Scale bar: 500 μm). (For interpretation of the references to colour in this figure legend, the reader is referred to the Web version of this article.)

New bone formation across the different groups was evaluated using H&E and Masson's trichrome staining (Fig. 6). In the control group, there were thin connective fibrous tissues in the defect sites, with no notable new bone formation observed at 6 weeks. In contrast, both the AS-GHF and AS-GHF + BMSCs groups displayed substantial collagen bundles and newly-formed bone extending inward from the periphery. Moreover, bone cavities and central canals have also formed within the emerging bone matrix in the AS-GHF and AS-GHF + BMSCs groups. In comparison, the AS-GHF + BMSCs group exhibited significantly greater bone volume compared to the AS-GHF group, which was corroborated by micro-CT measurements. By 12 weeks, all groups showed further bone repair; however, complete defect bridging with new bone was uniquely observed in the AS-GHF + BMSCs group, underscoring its superior reparative capability. Meanwhile, the hydrogels in the defect sites had nearly fully degraded, with only minimal Fe_3_O_4_ NPs remaining, suggesting that the degradation rate of the AS-GHF hydrogels was well-matched to the rate of new bone growth. Masson's trichrome staining showed that the control group had narrower and looser collagen fibrils, while the AS-GHF hydrogels exhibited improved collagen fibril deposition at 6 and 12 weeks. These newly formed mature collagen fibrils were significantly enhanced in the AS-GHF + BMSCs group, attributed to the synergistic effect of magnetic anisotropic hydrogels and BMSCs. These results highlight the positive role of AS-GHF + BMSCs in promoting collagen matrix development and new bone formation during the repair of critical-sized bone defects.

**Fig. 7:**
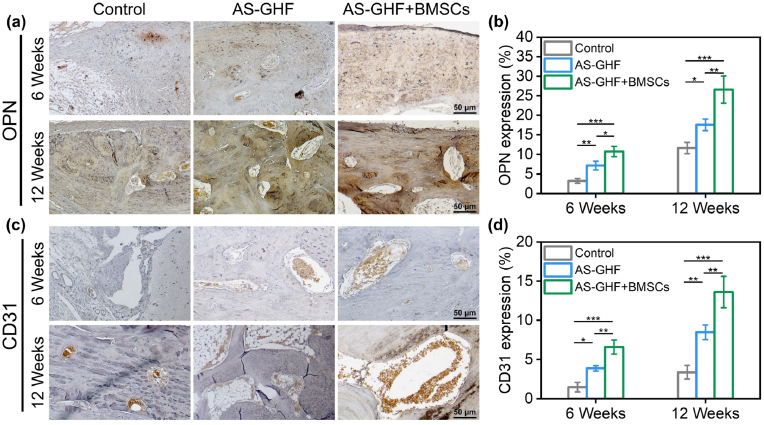
(**a-d**) Representative IHC images of OPN (**a**) and CD31 (**c**) with corresponding quantitative analyses (**b** and **d**) at 6- and 12-weeks post-operation. (n = 3, ∗p < 0.05, ∗∗p < 0.01, and ∗∗∗p < 0.001).

To verify the biomechanism of AS-GHF hydrogels combined with BMSCs in vivo, we conducted immunohistochemical (IHC) staining to evaluate the expression of the osteogenic marker osteopontin (OPN) and the angiogenic marker CD31 across different groups of bone defects. The analysis of OPN expression revealed significantly increased areas of OPN positivity in both the AS-GHF and AS-GHF + BMSCs groups (Fig. 7a). Quantitative assessments indicated that the AS-GHF + BMSCs group exhibited markedly higher OPN expression levels compared to the AS-GHF group at both week 6 (6.58 ± 0.91 % vs. 3.87 ± 0.34 %, p < 0.05) and week 12 (13.6 ± 2.01 % vs. 8.47 ± 0.92 %, p < 0.01) (Fig. 7b). These results suggest a synergistic enhancement of osteogenic protein expression facilitated by the AS-GHF hydrogel in conjunction with BMSCs. Moreover, CD31 IHC staining showed negligible CD31 positivity in the fibrous tissue of the bone defect within the blank group, while substantial CD31 expression was observed in both the AS-GHF and AS-GHF + BMSCs groups (Fig. 7c). Notably, the AS-GHF + BMSCs group demonstrated the highest levels of CD31 positivity along with an increased vascular diameter, as confirmed by quantitative analysis (Fig. 7d). Overall, the AS-GHF + BMSCs group exhibited significantly elevated expression levels of both OPN and CD31, highlighting its bioactivity to promote osteogenic and angiogenic expression.

It is noteworthy that our study primarily evaluated the promoting effect of AS-GH + BMSCs hydrogels on bone regeneration in the early stage (within 12 weeks), yet complete bone regeneration was not achieved. Despite radiographic and histological analyses confirming the formation of bony bridging, the structural maturity of the regenerated bone was significantly lower than that of native bone tissue. Therefore, we will conduct a long-term assessment of the bio-adaptability between material degradation kinetics and the rates of neo-bone remodeling, as well as the remodeling dynamics of nascent bone and its integration with host bone in future work.

## 4 Conclusion

In summary, we have developed anisotropic biomimetic 3D hydrogels for bone defect repair through a Liquid-in-Liquid bioprinting technique combined with magnetic induction. It was demonstrated that the hydrogen bonding and electrostatic interactions between Type A gelatin methacrylate and hyaluronic acid methacrylate resulted in bioinks with stable Liquid-in-Liquid bioprintability, which is crucial for the magnetically induced self-assembly alignment of Fe_3_O_4_ NPs within bioprinted constructs. The self-assembled Fe_3_O_4_ microfibers at an optimal concentration of 0.2 wt% displayed a uniform distribution within bioprinted hydrogels, with the lengths and widths measuring 178.64 ± 27.03 μm and 8.96 ± 1.49 μm, respectively. Furthermore, the BMSCs within magnetically bioprinted hydrogels exhibited high viability and proliferation activity. These cells were guided by the anisotropic Fe_3_O_4_ microfibers to adopt a highly elongated morphology, which activated key mechanical signaling pathways, such as Wnt, MAPK, PI3K-Akt and Calcium. Collectively, these pathways promoted the osteogenic differentiation of BMSCs. The magnetic anisotropic hydrogel containing BMSCs in a critical-sized bone defect model significantly facilitated the regeneration of bone tissue by promoting collagen matrix development and neovascularization. Overall, this highly customizable anisotropic biomimetic hydrogel represents a promising strategy for the personalized treatment of critical-sized bone defects.

## CRediT authorship contribution statement

**Rong Xu:** Writing – review & editing, Writing – original draft, Data curation. **Hua Zhang:** Writing – review & editing, Writing – original draft, Supervision, Resources, Data curation. **Yang Luo:** Methodology. **Shiyi Pan:** Formal analysis. **Chi Zhang:** Resources. **Xiaochuan Wu:** Supervision. **Guofeng Zhang:** Resources, Data curation. **Cuicui Su:** Writing – review & editing, Writing – original draft, Methodology. **Dongdong Xia:** Writing – review & editing, Writing – original draft, Data curation.

## Declaration of competing interest

The authors declare that they have no known competing financial interests or personal relationships that could have appeared to influence the work reported in this paper.

## Data Availability

Data will be made available on request.
