# Supplementary material for: Magnetically bioprinted anisotropic hydrogels promote BMSC osteogenic differentiation for bone defect repair
Source: Mater Today Bio. 2025 May 20;32:101885. doi: 10.1016/j.mtbio.2025.101885 (PMC12149643; doi:10.1016/j.mtbio.2025.101885)
Supplement: Multimedia component 1 [file mmc1.docx]

**Supporting Information**

**Magnetically Bioprinted Anisotropic Hydrogels Promote BMSC Osteogenic Differentiation for Bone Defect Repair**

Rong Xu^1,2^, Hua Zhang^2^*, Yang Luo^2^, Shiyi Pan^3^, Chi Zhang^1^, Xiaochuan Wu^1^, Guofeng Zhang^1^, Cuicui Su^2^*, Dongdong Xia^1^*

^1^ Department of Orthopedics, The First Affiliated Hospital of Ningbo University, Ningbo, Zhejiang, 315000, China

^2^ Research Institute of Smart Medicine and Biological Engineering, Health Science Center, Ningbo University, Ningbo, Zhejiang 315211, China

^3^ The Second Affiliated Hospital of Anhui Medical University, Hefei, Anhui, 230601, China

***Corresponding authors**: zhanghua@nbu.edu.cn (H.Z.); sucuicui@nbu.edu.cn (C.C.S.); drdongdongxia@163.com (D.D.X.)

**
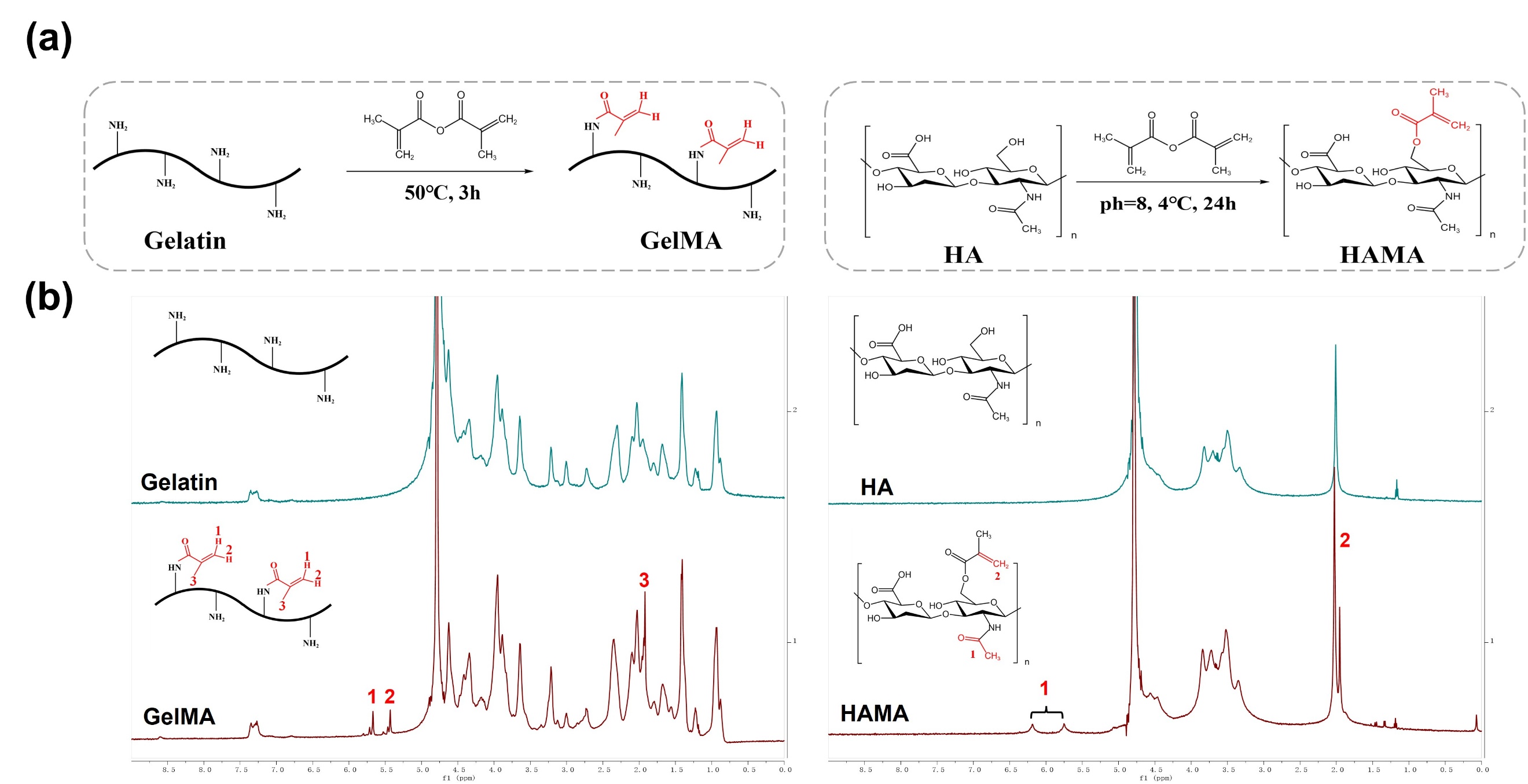
Figure S1.** Syntheses and characterization of GelMA and HAMA. (a) Schematic illustration of GelMA and HAMA syntheses. (b) ^1^H-NMR spectra of Gelatin, GelMA, HA and HAMA.


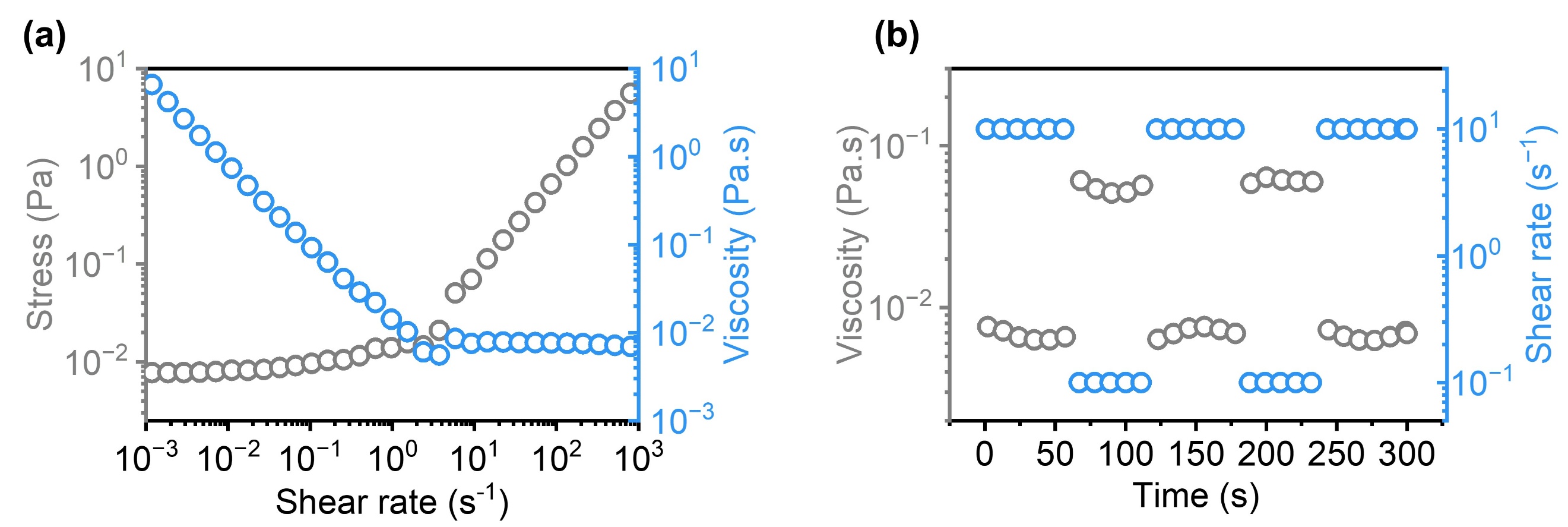


**Figure S2.** Rheological tests of 0.35% (wt/vol) *κ*-carrageenan supporting bath. (a) Shear-thinning and yield stress measurements on jammed *κ*-carrageenan bath via a shear ramp ranging from 0.001 to 1000 s^−1^ at 37°C. (b) Self-recovery analysis on *κ*-carrageenan bath upon continually cyclic shearing at alternate 10 s^−1^ and 0.1 s^−1^ rates at 37°C.


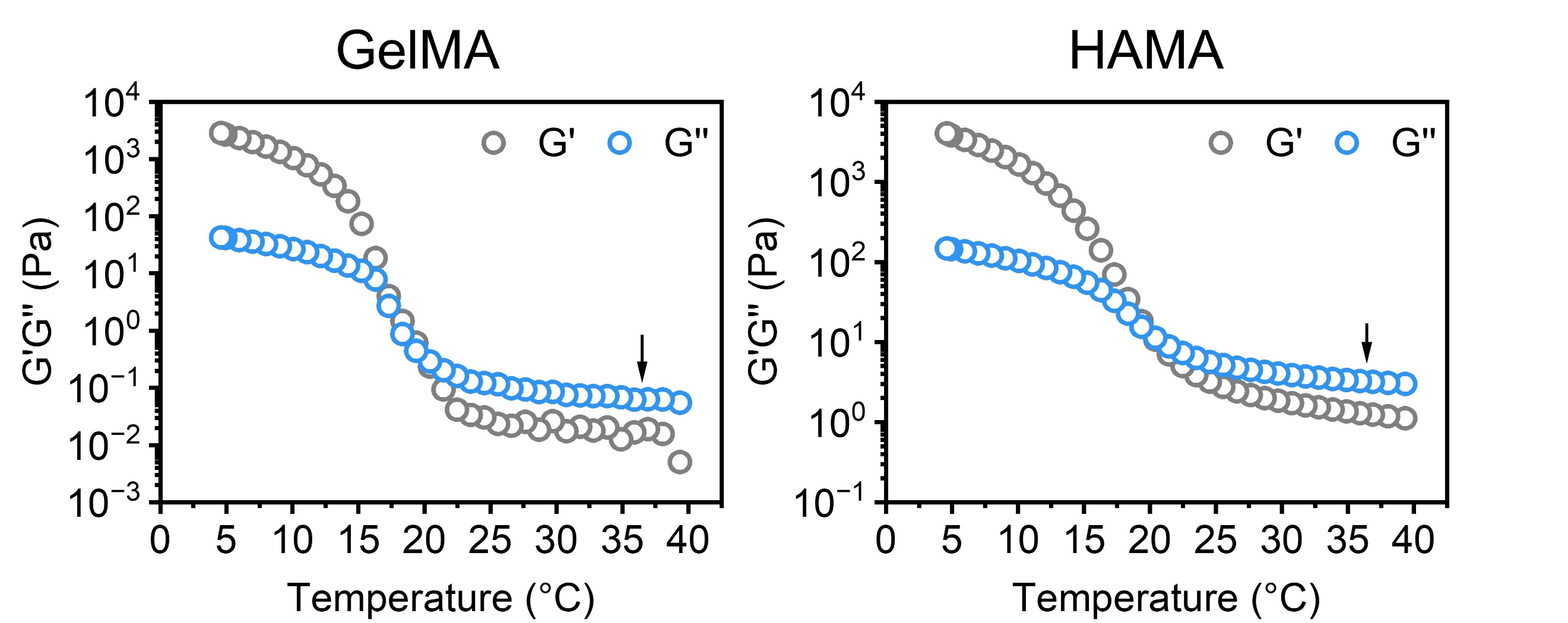


**Figure S3.** Thermo-stability measurement of GelMA and HAMA via temperature sweep from 5°C to 40°C.


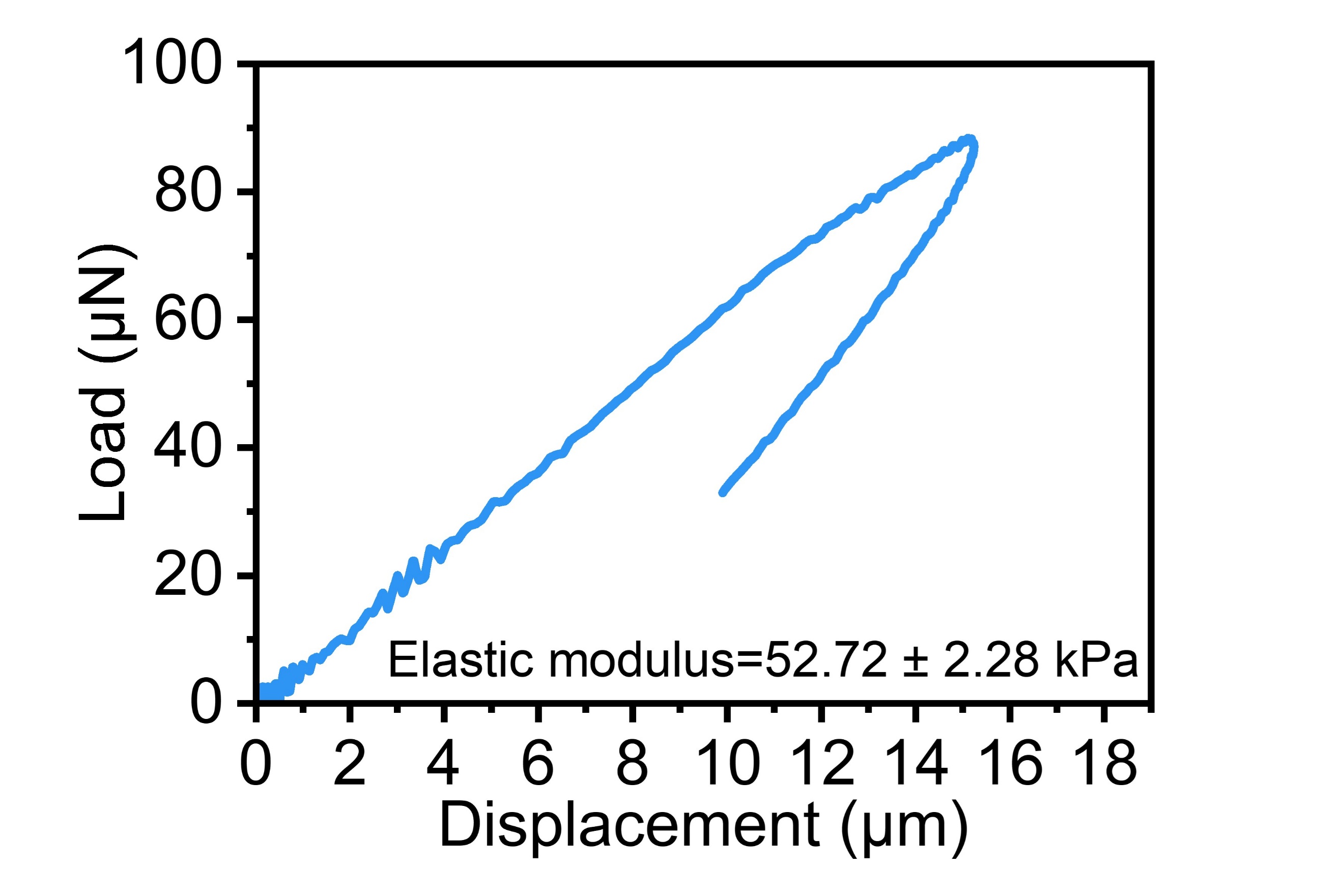


**Figure S4.** Nanoindention load-displacement curve of printed GelMA/HAMA hydrogels. (n=3).


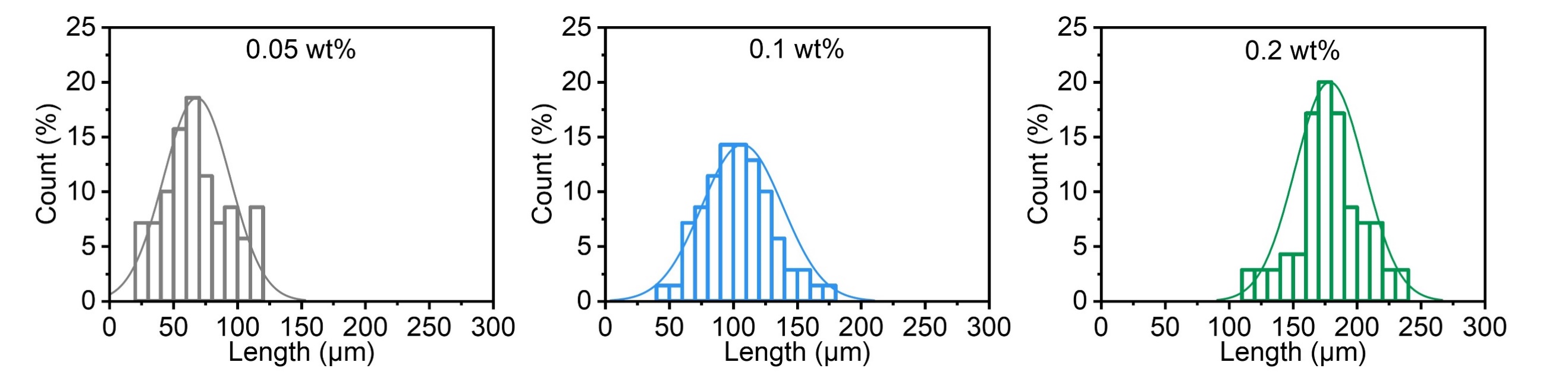


**Figure S5.** The length distribution of microfibers formed by different concentrations of Fe_3_O_4_ nanoparticles.


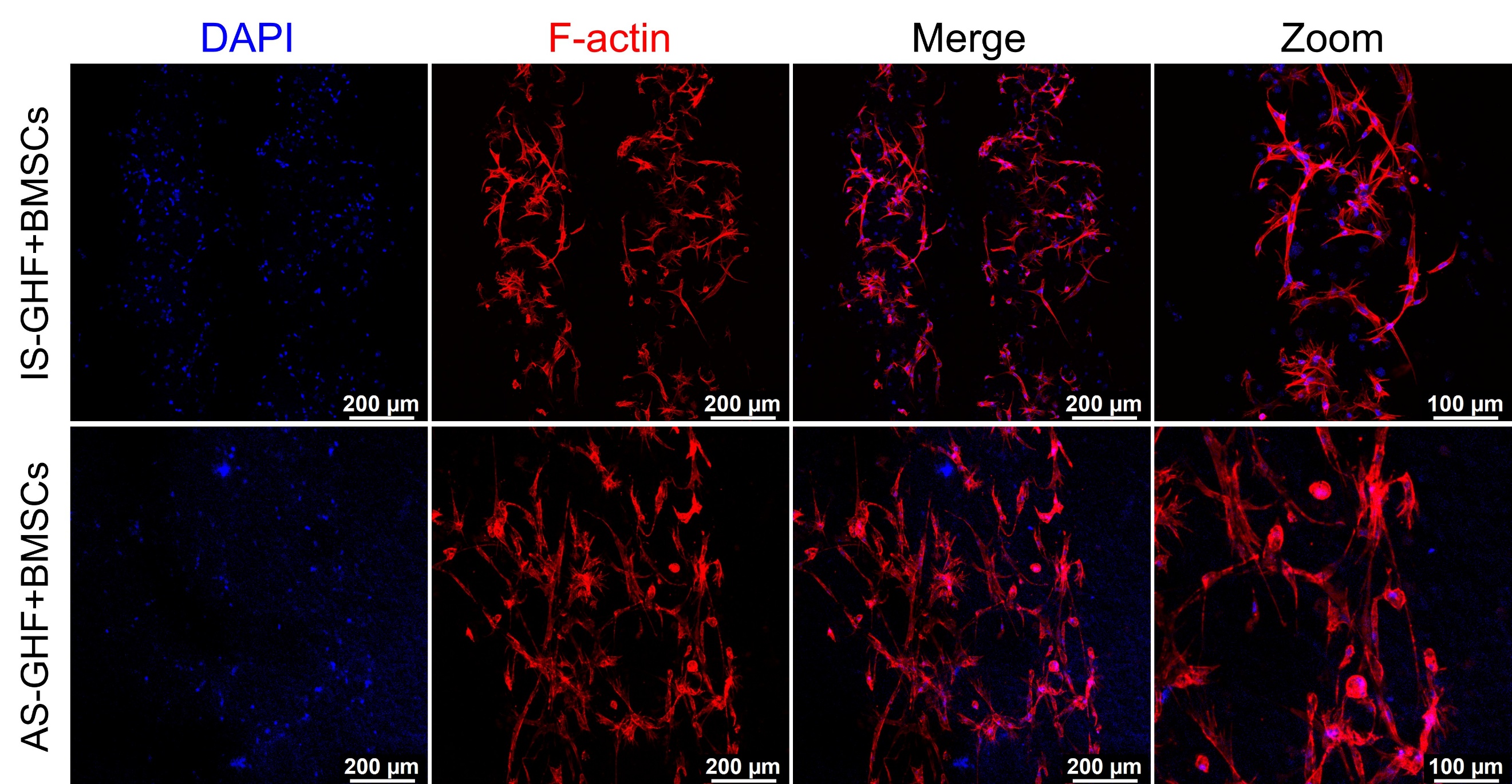


**Figure S6.** The representative cell morphology images of IS-GHF and AS-GHF were cultured in vitro for 3 days. (F-actin was stained red, and nuclei were stained blue).


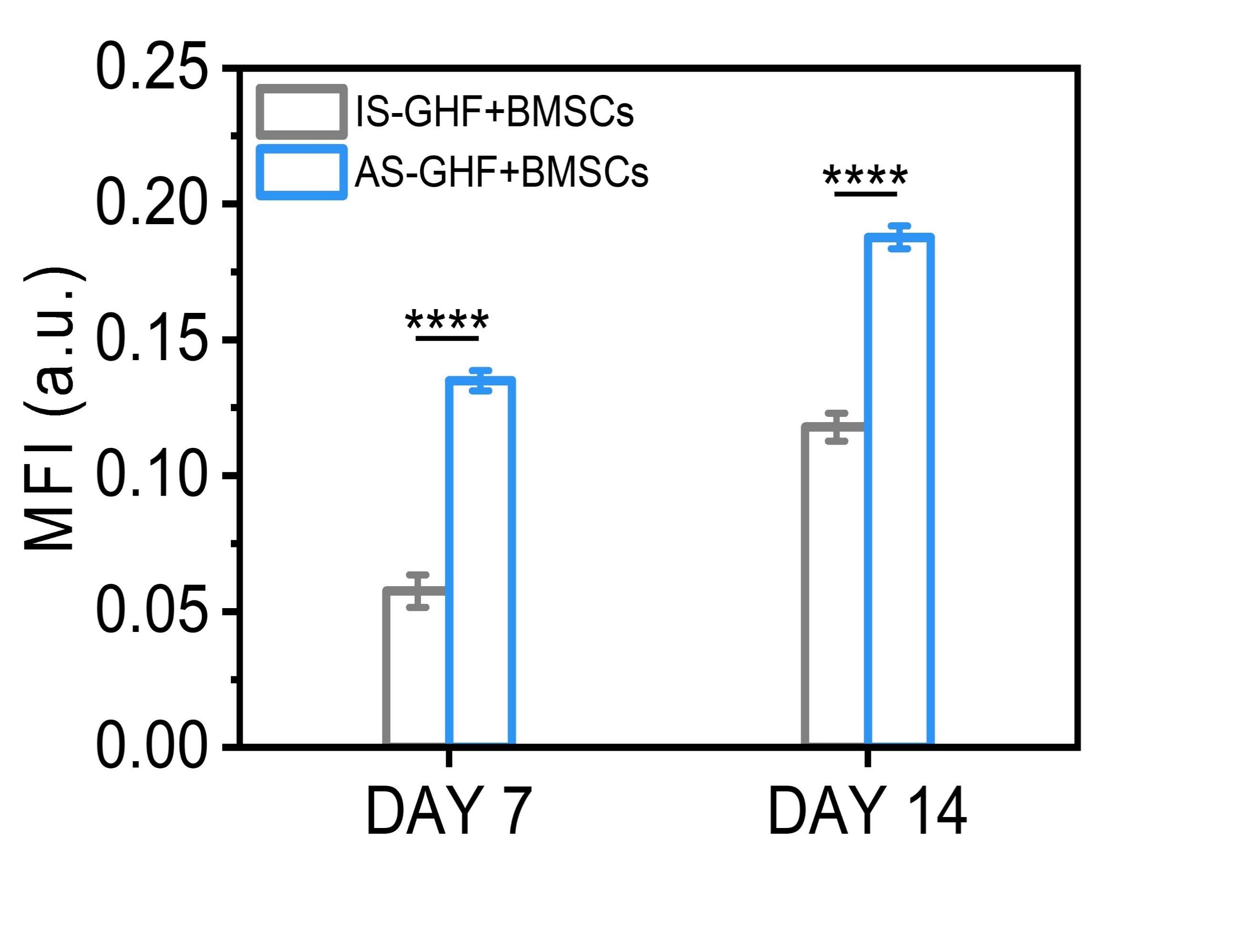


**Figure S7.** Quantitative analysis of the mean fluorescence intensity of immunofluorescence-stained images of OPN expression at 7 and 14 days. (n=3, ****p<0.0001).


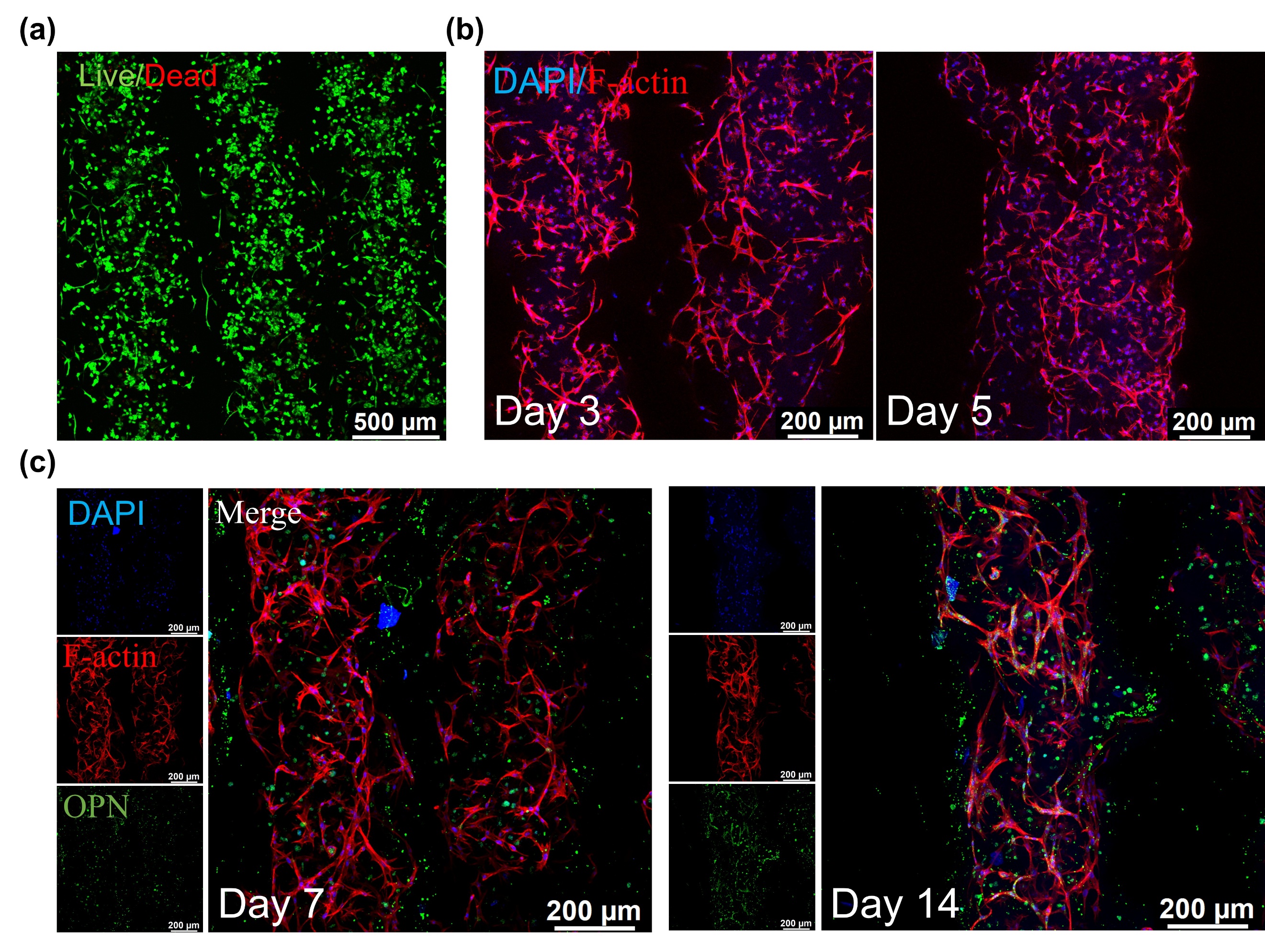


**Figure S8.** In vitro cytocompatibility, cell morphology analysis and osteogenic performance. (a) Representative Live/Dead staining images of BMSCs within GelMA/HAMA hydrogels with static magnetic field after 1 day of culture. (b) Representative images of BMSC morphologies within GelMA/HAMA hydrogels with static magnetic field following 3 and 5 days of culture. (c) Immunofluorescence staining images of osteopontin (OPN) after 7 and 14 days of osteogenic induction.


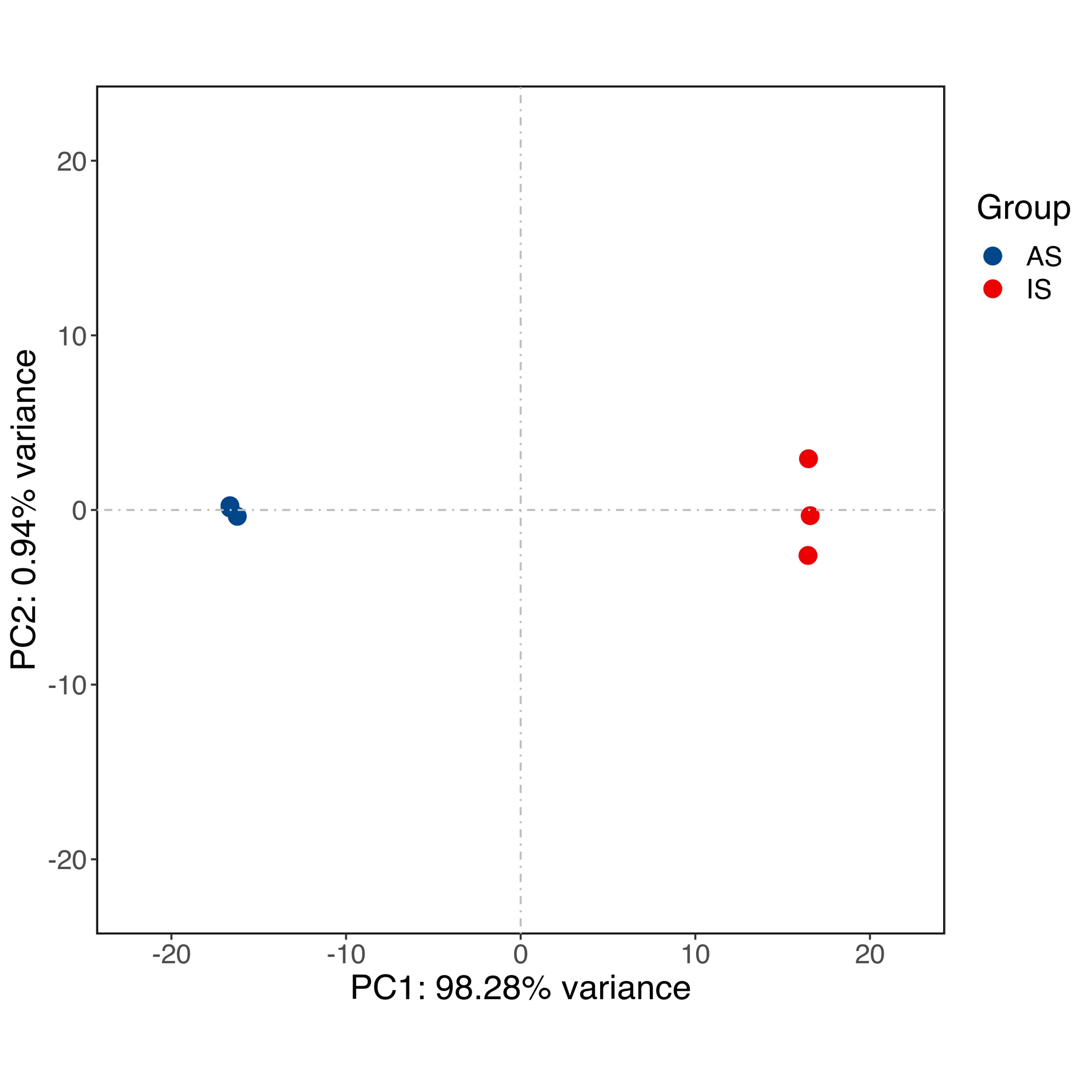


**Figure S9.** The PCA results of IS-GHF and AS-GHF hydrogels.


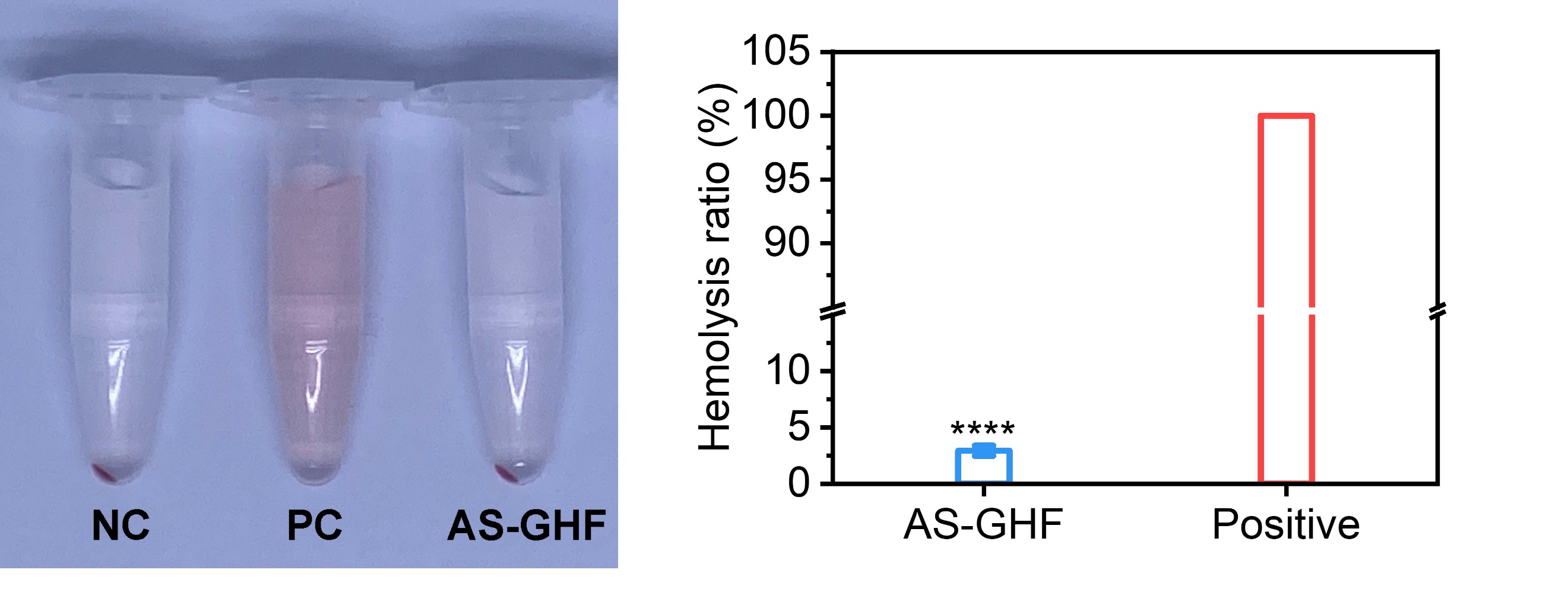


**Figure S10.** Hemolysis rate upon incubation with AS-GHF, PC (positive control), and NC (negative control). (n=3, ****p<0.0001).


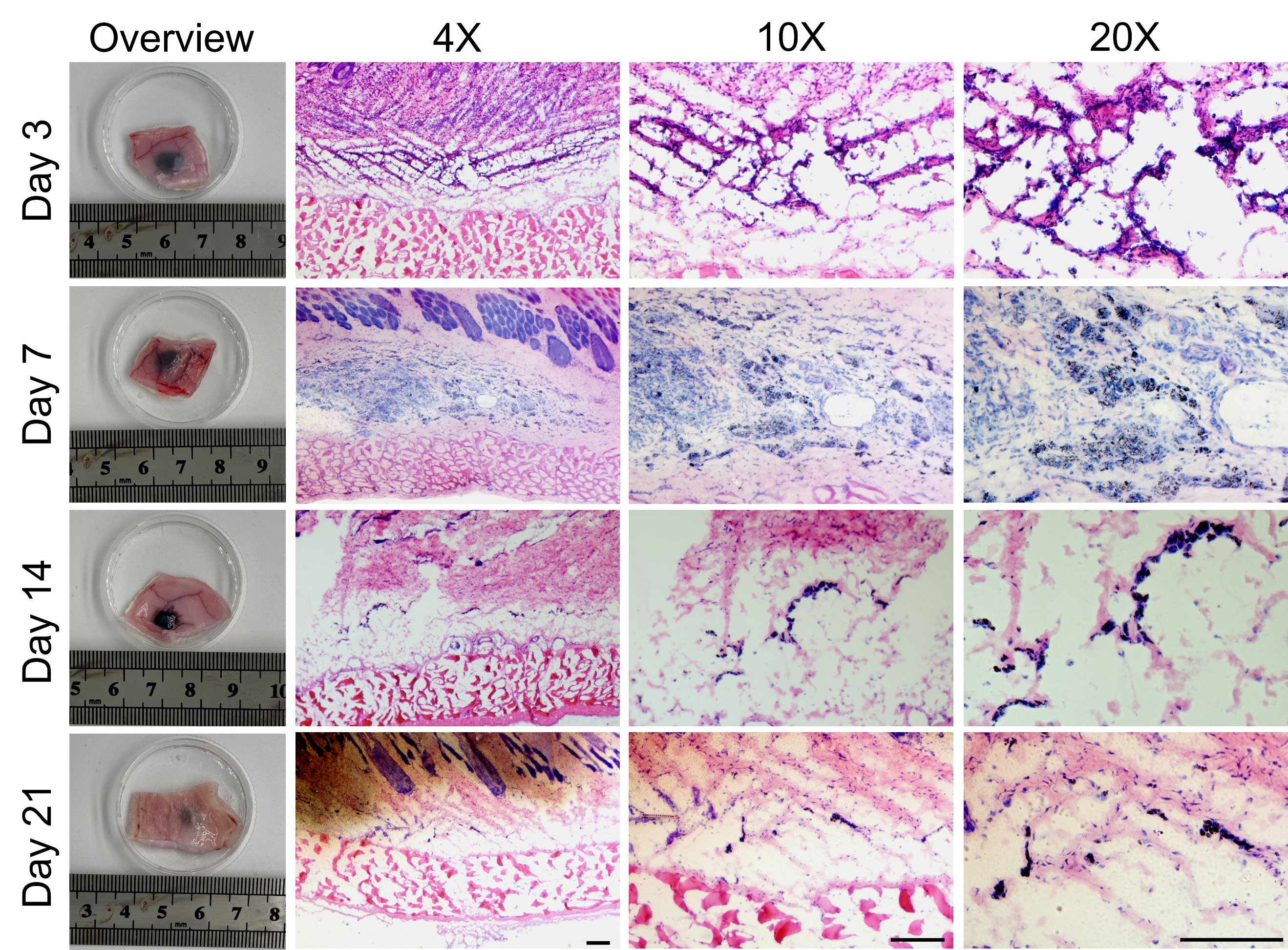


**Figure S11.** In vivo biocompatibility and degradation of AS-GHF hydrogel. H&E staining images of corresponding skin tissues at 3, 7, 14 and 21 days after implantation. (Scale bar: 200 μm).


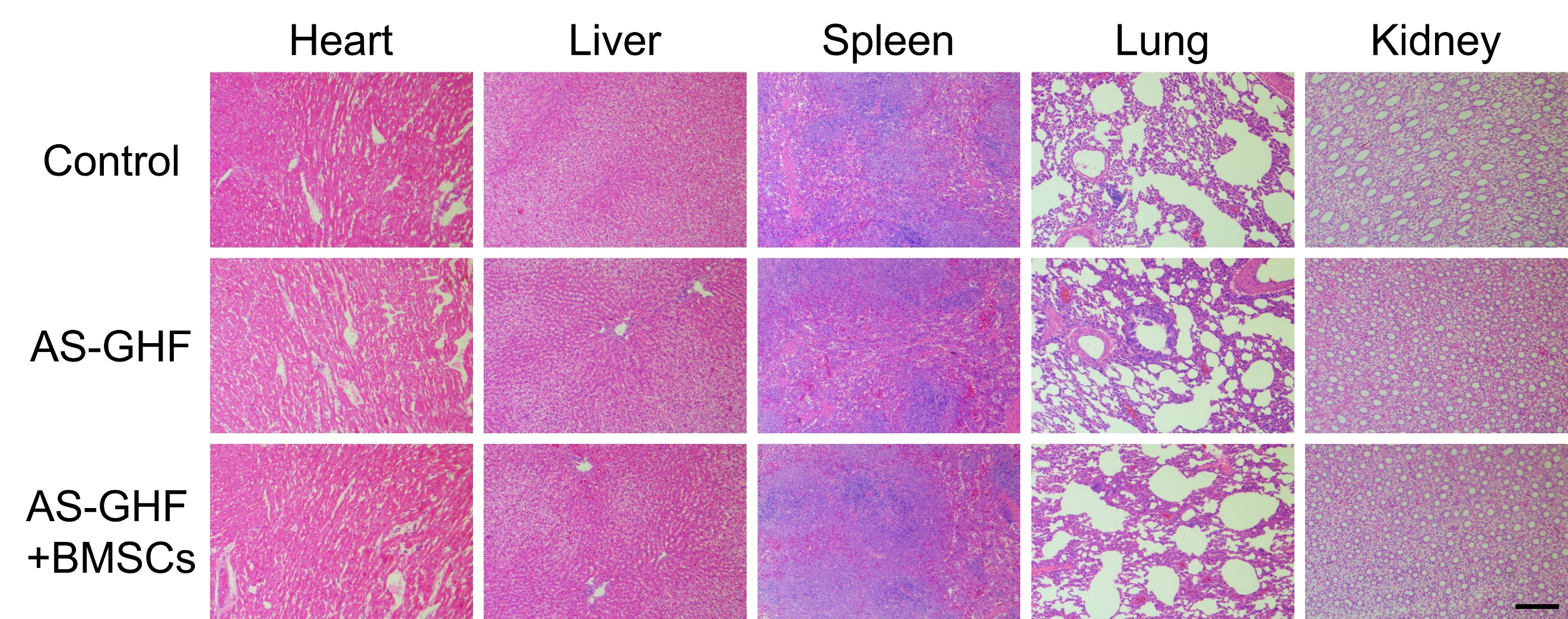


**Figure S12.** H&E staining images of rabbit heart, liver, spleen, lung, and kidney at 12 weeks after operation. (Scale bar: 100 μm).

**Video S1.** The fluidity of *κ*-carrageenan bath at 37°C.

**Video S2.** The process of self-assembly of Fe_3_O_4_ nanoparticles into aligned microfibers under magnetic field.
